# Elucidating Microstructural Alterations in Neurodevelopmental Disorders: Application of Advanced Diffusion‐Weighted Imaging in Children With Rasopathies

**DOI:** 10.1002/hbm.70087

**Published:** 2024-12-12

**Authors:** Julia R. Plank, Elveda Gozdas, Erpeng Dai, Chloe A. McGhee, Mira M. Raman, Tamar Green

**Affiliations:** ^1^ Division of Interdisciplinary Brain Sciences, Department of Psychiatry and Behavioral Sciences Stanford University Palo Alto California USA; ^2^ Department of Radiology Stanford University Stanford California USA

**Keywords:** diffusion kurtosis imaging, diffusion‐weighted imaging, magnetic resonance imaging, neurite orientation density and dispersion imaging, neurodevelopmental disorders, Rasopathies

## Abstract

Neurodevelopmental disorders (NDDs) can severely impact functioning yet effective treatments are limited. Greater insight into the neurobiology underlying NDDs is critical to the development of successful treatments. Using a genetics‐first approach, we investigated the potential of advanced diffusion‐weighted imaging (DWI) techniques to characterize the neural microstructure unique to neurofibromatosis type 1 (NF1) and Noonan syndrome (NS). In this prospective study, children with NF1, NS, and typical developing (TD) were scanned using a multi‐shell DWI sequence optimized for neurite orientation density and dispersion imaging (NODDI) and diffusion kurtosis imaging (DKI). Region‐of‐interest and tract‐based analysis were conducted on subcortical regions and white matter tracts. Analysis of covariance, principal components, and linear discriminant analysis compared between three groups. 88 participants (*M*_age_ = 9.36, SD_age_ = 2.61; 44 male) were included: 31 NS, 25 NF1, and 32 TD. Subcortical regions differed between NF1 and NS, particularly in the thalamus where the neurite density index (NDI; estimated difference 0.044 [95% CI: −0.034, 0.053], *d* = 2.36), orientation dispersion index (ODI; estimate 0.018 [95% CI: 0.010, 0.026], *d* = 1.39), and mean kurtosis (MK; estimate 0.049 [95% CI: 0.025, 0.072], *d* = 1.39) were lower in NF1 compared with NS (all *p* < 0.0001). Reduced NDI was found in NF1 and NS compared with TD in all 39 white matter tracts investigated (*p* < 0.0001). Reduced MK was found in a majority of the tracts in NF1 and NS relative to TD, while fewer differences in ODI were observed. The middle cerebellar peduncle showed lower NDI (estimate 0.038 [95% CI: 0.021, 0.056], *p* < 0.0001) and MK (estimate 0.057 [95% CI: 0.026, 0.089], *p* < 0.0001) in NF1 compared to NS. Multivariate analyses distinguished between groups using NDI, ODI, and MK measures. Principal components analysis confirmed that the clinical groups differ most from TD in white matter tract‐based NDI and MK, whereas ODI values appear similar across the groups. The subcortical regions showed several differences between NF1 and NS, to the extent that a linear discriminant analysis could classify participants with NF1 with an accuracy rate of 97%. Differences in neural microstructure were detected between NF1 and NS, particularly in subcortical regions and the middle cerebellar peduncle, in line with pre‐clinical evidence. Advanced DWI techniques detected subtle alterations not found in prior work using conventional diffusion tensor imaging.

## Graphical abstract

Differences in brain microstructure between neurodevelopmental disorders neurofibromatosis type‐1 (NF1) and Noonan syndrome (NS)were detected using neurite orientation density and dispersion imaging and diffusion‐kurtosis imaging. Differential decreases in neurite density (NDI) and mean kurtosis (MK) were found in each condition compared to age and sex matched typically developing (TD), particularly in the middle cerebellar peduncle.

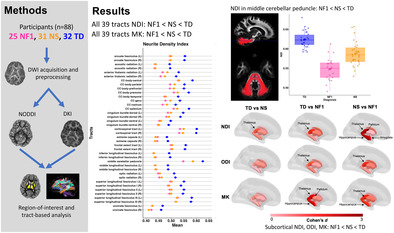

## 1 Introduction

Neurodevelopmental disorders (NDDs) can have severe impacts on functioning, yet treatment options are limited (Homberg et al. 2016). Development of appropriate treatments is hindered by the heterogeneity of NDDs and the prevalence of comorbidities (Parenti et al. 2020). Elucidating the neurobiological mechanisms underlying NDDs is crucial for the identification of targets for treatment. Many NDDs have a genetic basis, therefore neuroimaging studies with a “genetics‐first” approach may provide insight into the neural structure while limiting the heterogeneity that hinders conclusions. While extensive animal research supports this approach (Homberg et al. 2016), research on human clinical populations is lacking.

Rasopathies are NDDs caused by genetic mutations in the Ras/ mitogen activated protein kinase (Ras‐MAPK) signaling pathway (Tidyman and Rauen 2016). Neurofibromatosis type 1 (NF1) and Noonan syndrome (NS) are common Rasopathies with well‐established genetic origins. Mutations in *PTPN11*, *LZTR1*, *KRAS*, *SOS1*, or *RAF1* genes cause NS, whereas a mutation in *NF1* causes NF1 (Tidyman and Rauen 2016). Autism spectrum disorder, attention‐deficit hyperactivity disorder (ADHD), and oppositional defiant disorder are frequent comorbidities (Garg et al. 2017; Naylor et al. 2023); with several genes implicated in NS also associated with autism spectrum disorder (Pinto et al. 2014). Preclinical studies of NF1 and NS indicate pronounced neural alterations unique to each condition (Kang and Lee 2019), however, clinical neuroimaging studies in affected individuals suggest similar aberrations (Siqueiros‐Sanchez et al. 2024). Neuroimaging methods with greater specificity are needed to elucidate differences, should they exist, between the conditions in clinical populations with implications for treatment and translation.

The clear genetic origins of NF1 and NS enable us to gain insights at the cellular level from mouse models of these conditions. In NF1, the *NF1* gene is inactivated which leads to a reduction in neurofibromin expression. Neurofibromin is believed to function as a negative growth regulator for astrocytes, and thus astrocyte production is among the cellular consequences of the *NF1* mutation (Gutmann et al. 1999). Neuron formation, on the other hand, tends to be downregulated in NF1 (Hegedus et al. 2007). In NS, mutations such as *PTPN11*, which encode the protein SHP2, stabilize SHP2 in the active conformation such that the Ras‐MAPK pathway is consistently activated. The downstream cellular effects include increased neurogenesis and a reduction in astrocyte formation (Gauthier et al. 2007; Ehrman et al. 2014). Additionally, there is evidence in NS of activated inflammatory microglia, which may play a role in the inhibition of astrocyte formation (Antony 2011).

Recent advances in MRI techniques may allow a more specific revealing of underlying neural microstructure in NF1 and NS. Diffusion‐weighted imaging (DWI) techniques are uniquely sensitive to cellular architecture (Alexander et al. 2019). Neurite orientation density and dispersion imaging (NODDI) and diffusion kurtosis imaging (DKI) are two such techniques that enable insights into features of tissue at the microscopic level. Prior work shows that NODDI and DKI are sensitive to alterations in glial cell density (Yi et al. 2019; Zhuo et al. 2012). For example, the orientation dispersion index (ODI) derived from NODDI has previously demonstrated sensitivity to microglial proliferation (Yi et al. 2019), while the mean kurtosis (MK) measure derived from DKI was associated with astrocyte proliferation (Zhuo et al. 2012). NODDI and DKI show promise for the detection of distinct patterns of glial cell alterations evident in NF1 and NS.

To our knowledge, advanced DWI techniques such as NODDI and DKI have not yet been applied across the brain to clinical populations with NF1 and NS. A previous study of 17 participants with NF1 examined T2‐hyperintensities using NODDI and DKI, however, they did not compare to controls nor to another clinical syndrome (Billiet et al. 2014). NODDI and DKI were applied previously to NDDs such as autism spectrum disorder and ADHD (Kraguljac et al. 2023; Connaughton et al. 2022). The results, however, do not indicate a clear pattern across studies. Our study aims to explore the potential of a genetics‐first approach in conjunction with advanced diffusion techniques to identify more consistent patterns. Through our genetics‐first approach, we can use findings from animal research on NF1 and NS to draw inferences about the microstructural basis of our imaging findings. Successful identification of microstructural differences between NS and NF1 using these diffusion models would suggest that NODDI and DKI may be useful tools for elucidating the pathophysiology underlying further NDDs.

We investigated the potential of NODDI and DKI to elucidate microstructural alterations in NF1 and NS. We hypothesized that alterations to NODDI and DKI would yield disparate findings in NF1 and NS consistent with pre‐clinical models. For example, results would reflect the promotion of gliogenesis and decreased neurogenesis in NF1, and the opposite in NS (Gutmann et al. 1999; Hegedus et al. 2007; Gauthier et al. 2007; Ehrman et al. 2014; Antony 2011). Identification of differences between NF1 and NS would suggest a genetics‐first approach in conjunction with advanced DWI may assist in understanding the neurobiology of NDDs and development of new treatments. A better understanding of the distinct effects of Ras‐MAPK mutations on the brain, and the ability to non‐invasively image these manifestations, would be of value for future clinical trials aiming to develop targeted treatments and improve treatment efficacy in these populations.

## 2 Materials and Methods

### 2.1 Participants

For this prospective study, participants with NF1 and NS were recruited from May 2021 through December 2023 across the United States and Canada. Typical developing (TD) participants were recruited using local networks and social media advertising limited to the San Francisco Bay area. Sixty‐six members of the participant population were reported previously (NF1 *n* = 20, NS *n* = 22, TD *n* = 26) (Siqueiros‐Sanchez et al. 2024). Proof of genetic testing was required for individuals with NF1 or NS to demonstrate the presence of appropriate mutations. The inclusion criteria for all NF1 and NS participants are as follows (a) age from 5 years, 0 months to 17 years, 11 months; (b) intelligence quotient (IQ) criterion ≥ 70; and (c) gestational age > 34 weeks. Participants with NF1 and focal areas of signal intensity with no mass effect and/or low‐grade optic pathway glioma were not excluded. TD participants were group‐matched to participants with NF1 and NS based on age, sex, handedness, socioeconomic status, pubertal status, and ethnicity. For all participants, the exclusion criteria includes (a) presence of neurological or psychiatric disease (e.g., psychotic symptoms); (b) sensory deficits that would preclude participation in assessments or imaging; (c) history of significant head trauma with loss of consciousness; (d) use of psychotropic medications or currently taking medications with central nervous system effects within 5 half‐lives of the scan; (e) contraindications to MRI (e.g., metal implants, orthodontia, claustrophobia); (f) history of alcohol or drug use; (g) premature birth (< 34 weeks); (h) low birth weight (< 2000 g); (i) history of head trauma with loss of consciousness; neurological disorders known to affect cognitive development or brain structure; (j) known presence of gliomas in cerebellum, brainstem, or basal ganglia; and (k) contraindications to MRI. Additionally, participants with NF1 were excluded if they received an MEK inhibitor or chemotherapy, and/or show presence of cerebellar, brainstem, tectal plate, and basal ganglia gliomas. Participants with NF1 and NS are not excluded if they show clinical symptoms associated with typical syndromic cognitive‐behavioral features (e.g., inattention, anxiety, learning disorder, or autistic traits). Pubertal status was determined using Tanner staging (Marshall and Tanner 1970, 1969). A full‐scale IQ ≥ 70, acquired using the Wechsler Abbreviated Scale of Intelligence 2nd Edition, was required to maximize study compliance (Wechsler 1999).

Legal guardians provided written informed consent and participants aged over 7 years provided complementary written assent. The Stanford University School of Medicine Institutional Review Board approved all procedures in this work involving human subjects. All procedures comply with the ethical standards of the national and institutional committees on human experimentation and with the Helsinki Declaration of 1975, as revised in 2008. Eligible participants in this study included 32 children with NF1, 35 with NS, and 36 TD.

### 2.2 Imaging Protocol

The MRI scan was completed on a GE Premier 3.0 Tesla whole‐body system using a standard 48‐channel head coil (GE Healthcare, Milwaukee, WI). Structural data were collected using a whole‐brain high‐resolution T1‐weighted magnetization‐prepared rapid gradient‐echo (MPRAGE) sequence. DWI data were collected using a multi‐shell acquisition with *b* = 500 s/mm^2^ (6 directions), *b* = 1000s/mm^2^ (15 directions), *b* = 2000s/mm^2^ (15 directions), and *b* = 3000 s/mm^2^ (60 directions). Further parameters are detailed in the Data S1. DWI data were preprocessed (author MR) using FSL 6.0.5 (FMRIB Analysis Group, Oxford, UK), with *topup* susceptibility‐induced distortion correction and *eddy* for correction of eddy currents‐induced distortions and subject movements (Andersson and Sotiropoulos 2016; Andersson, Skare, and Ashburner 2003).

### 2.3 Image Analysis

The data analysis pipeline is visualized in Figure S1. NODDI is a multi‐compartment biophysical model, which models the diffusion signal as a combination of three compartments: cerebrospinal fluid, extracellular, and intracellular, represented by the free water volume fraction, orientation dispersion index (ODI), and neurite density index (NDI), respectively (Zhang et al. 2012; Kamiya, Hori, and Aoki 2020). NDI estimates the density of axons and dendrites whereas ODI represents their orientational coherence (Zhang et al. 2012). NODDI can be applied to both grey and white matter. In white matter, higher values of NDI represent greater density of axons, whereas higher values of ODI indicate greater axon fanning and bending. In grey matter, elevated NDI indicates decreased density of dendrites, whereas higher ODI suggests more dispersion of dendrites. The NODDI maps were generated using the NODDI Matlab Toolbox v. 1.05 (UCL, UK) through fitting to the preprocessed diffusion data in MATLAB R2023b (Mathworks, Natick, MA).

DKI models the diffusion‐weighted signal in a similar manner to the diffusion tensor, but with an additional term *K* to account for the kurtosis of the diffusion distribution (Wu and Cheung 2010). DKI sequences utilize high *b* values to probe the complex neural microstructure and quantify the degree of diffusion restriction. The DKI maps for MK were generated using the *dtifit* command in FSL by including the *kurtdir* argument for multi‐shell data (Jbabdi et al. 2012). MK is the average kurtosis in all directions; a higher value indicates greater barriers to diffusion. Similar kurtosis values have been found in white matter and subcortical regions, possibly because both contain myelinated neurons (Maiter et al. 2021). The maps for each participant were quality checked prior to analysis (JP) (Figure S2).

Average values of NDI, ODI, and MK were extracted from each of the following six regions‐of‐interest (ROIs): amygdala, caudate, hippocampus, pallidum, putamen, and thalamus. These ROIs comprise grey and white matter, whereas the tracts are predominantly white matter. ROIs and white matter tracts were selected based on prior diffusion tensor imaging studies where significant alterations to parameters such as fractional anisotropy and mean diffusivity were found in NF1 and NS (Siqueiros‐Sanchez et al. 2024; Fattah et al. 2021; Karlsgodt et al. 2012; Tam et al. 2021). TRActs Constrained by UnderLying Anatomy (TRACULA) delineated 42 major white matter tracts using probabilistic tractography (Yendiki et al. 2011). Further details are in the Data S1.

### 2.4 Statistical Analysis

For each ROI and white matter tract, an analysis of variance was used to assess between‐group differences including age and sex as covariates. The presence of T2‐hyperintensities was included as an additional covariate in analysis of subcortical ROIs. Post hoc analysis was conducted using independent samples pairwise *t‐*tests corrected using the Tukey–Kramer test for unequal groups. Data were visually examined for approximate normality. The Bonferroni correction was applied to address the issue of multiple comparisons. Bonferroni corrected *p* values < 0.05 were considered statistically significant. At least 18 subjects were required in each group to meet a minimum detectable effect size of *d* = 0.88, as calculated based on prior diffusion tensor imaging studies on NS and NF1 (Fattah et al. 2021; Tam et al. 2021). Further details are available in the Data S1. Statistical analysis was completed by JP in R 4.3.1 (R Core Team, 2023).

Multivariate approaches were used to complement our univariate results. For tract‐based data, we used principal components analysis (PCA) to extract features and reduce dimensionality. PCA is a data‐driven technique designed to capture the most significant sources of variability in the data. The weights of the loadings in the principal components may also reveal the patterns or combinations of variables that contribute most to the variability in the data (feature extraction). Prior to PCA, missing data were imputed using a predictive mean matching algorithm performed by the *Multiple Imputation by Chained Equations* (*MICE*) package in R. 2.92% of data were imputed. The data were then normalized and PCA was conducted using the *prcomp* function in R, which performs PCA using the singular value decomposition approach.

Univariate results suggested microstructural alterations to subcortical regions may distinguish NS and NF1, therefore subcortical diffusion parameters were entered into a linear discriminant analysis. Linear discriminant analysis determines the extent to which two or more groups can be separated by maximizing the ratio of between‐class variance to the within‐class variance. This analysis may identify key subcortical regions or diffusion parameters that demonstrate microstructural differences between NF1 and NS. Linear discriminant analysis was used to predict the probability of belonging to a group (TD, NF1, NS) based on continuous predictor variables (NDI, ODI, MK). The data were centered and linear discriminant analysis was then conducted using the *lda* function within the MASS package in R. Leave‐one‐out cross‐validation was used to test the linear discriminant functions. Leave‐one‐out cross‐validation is performed by using all the observations except the *n*th to construct the discriminant function, and then said discriminant function is used to classify the *n*th observation. This procedure is repeated for all observations in the dataset.

## 3 Results

### 3.1 Participant Characteristics

**FIGURE 1:**
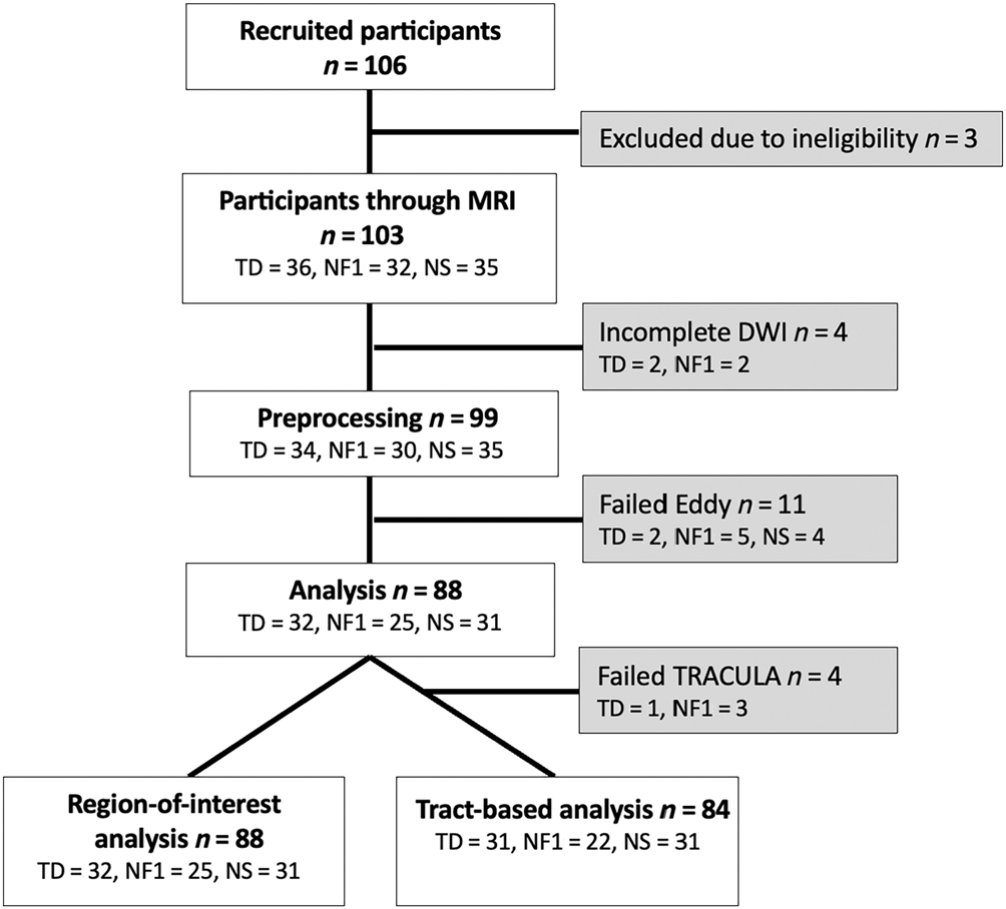
Flowchart of participants through the study. NF1 = neurofibromatosis type 1; NS = Noonan syndrome; TD = typical developing.

A total of 88 subjects (*M*_age_ = 9.36, SD_age_ = 2.61; 44 male) were included in analysis (Table 1). The flow of participants through the study are shown in Figure 1. There were 25 participants in the NF1 group (*M*_age_ = 9.20, SD_age_ = 2.27; 15 male); 31 in the NS group (*M*_age_ = 10.0, SD_age_ = 3.22; 12 male); and 32 in the TD group (*M*_age_ = 8.88, SD_age_ = 2.13; 15 male). Details on participant mutations are in the Data S1. The groups did not differ significantly by age (*p* = 0.090), sex (*p* = 0.259), or pubertal stage (*p* > 0.05) (Table S1). Significantly lower scores were found for clinical groups compared with TD on all IQ measures (*p* < 0.010). Results of the sensitivity analysis for T2‐hyperintensities are shown in Table S2.

**TABLE 1:** Demographics and descriptive statistics of included participants.

	All (*n* = 88)	TD (*n* = 32)	NF1 (*n* = 25)	NS (*n* = 31)
Age	9.36 (2.61)	8.88 (2.13)	9.20 (2.27)	10.0 (3.22)
Sex (M/F)	44/44	17/15	15/10	12/19
FSIQ	104 (15.4)	115 (12.9)	96.5 (13.3)	98.5 (13.2)
VIQ	106 (16.5)	116 (13.1)	101 (17.5)	102 (15.0)
PIQ	101 (18.8)	110 (17.0)	92.5 (11.5)	98.7 (21.6)
Tanner 1‐Stage 1/2/3/4	43/19/14/6	15/11/4/1	9/6/5/3	19/2/5/2
Tanner 2‐Stage 1/2/3/4	58/10/11/4	20/7/3/1	15/3/5/0	23/0/3/3
Stimulant	15	0	9	6
Growth hormone	8	0	0	8

*Note:* Data presented as counts or mean (SD). Tanner 1 refers to Tanner Pubic Hair Scale. Tanner 2 refers to Female Breast Development/Male External Genitalia Scale. Tanner Scales exclude 1 TD, 2 NF1, 2 NS.

Abbreviations: ANOVA = analysis of variance; FSIQ = Full‐Scale Intelligence Quotient; NF1 = neurofibromatosis type 1; NS = Noonan syndrome; PIQ = Performance Intelligence Quotient; TD = typical developing; VIQ = Verbal Intelligence Quotient.

### 3.2 ROI Analysis of NDI, ODI, and MK

**FIGURE 2:**
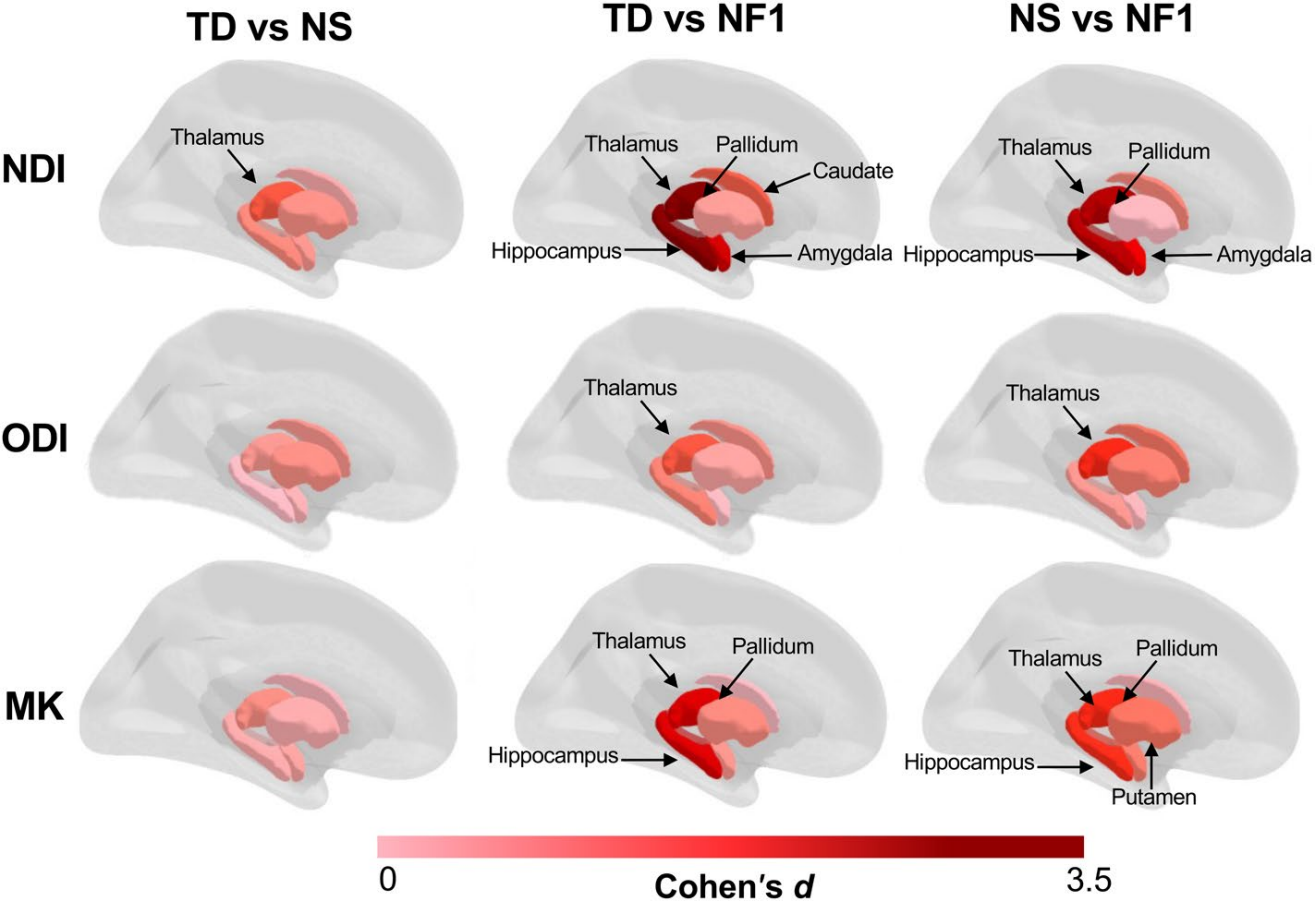
Effect sizes in subcortical ROIs for NDI, ODI, and MK between groups. ROIs with a large effect size (Cohen's *d* ≥ 0.80) are labelled. Effect sizes were converted to absolute values for purpose of visualization. Visualization created using *ggseg3d* package in R 4.3.1 (R Core Team, 2023). MK = mean kurtosis; NDI = neurite density index; NF1 = neurofibromatosis type 1; NS = Noonan syndrome; ODI = orientation dispersion index; TD = typical developing.

In subcortical regions, the NDI results showed a trend of NF1 < NS < TD (Table 2). Lower NDI values were found in NF1 and in NS compared to TD (Figures 2 and S3). NDI was lower in NF1 compared with NS in the amygdala, hippocampus, pallidum, and thalamus (all *p* < 0.0001), with the largest effect observed in the thalamus (an estimated difference of 0.044 [95% CI: 0.034, 0.053], *d* = 2.36). Overall, NDI detected the greatest number of differences between groups and therefore showed the most sensitivity of the three diffusion measures investigated.

**TABLE 2:** Confidence intervals from analysis of subcortical regions.

		TD—NF1				TD—NS				NF1—NS			
	ROI	Estimate (95% CI)	p	p (corr)	d	Estimate (95% CI)	p	*p* (corr)	d	Estimate (95% CI)	p	*p* (corr)	d
NDI	Amygdala	0.040 (0.028, 0.051)	**< 0.0001**	**< 0.0001**	**1.97**	0.009 (−0.001, 0.020)	0.105	0.129	0.45	−0.030 (−0.042, −0.019)	**< 0.0001**	**< 0.0001**	**−1.66**
	Caudate	0.025 (0.009, 0.040)	**0.0001**	**0.0008**	**1.00**	0.012 (−0.003, 0.026)	0.134	0.158	0.22	−0.013 (−0.028, 0.003)	0.134	0.156	−0.61
	Hippocampus	0.061 (0.050, 0.072)	**< 0.0001**	**< 0.0001**	**3.34**	0.012 (0.002, 0.023)	**0.015**	**0.021**	**0.59**	−0.048 (−0.060, −0.037)	**< 0.0001**	**< 0.0001**	**−2.34**
	Pallidum	0.098 (0.064, 0.132)	**< 0.0001**	**< 0.0001**	**1.41**	0.034 (0.002, 0.066)	**0.035**	**0.039**	**0.27**	−0.064 (−0.098, −0.030)	**< 0.0001**	**< 0.0001**	**−0.98**
	Putamen	0.007 (−0.004, 0.018)	0.315	0.437	0.37	0.014 (0.003, 0.025)	**0.007**	**0.007**	**0.50**	0.007 (−0.005, 0.018)	0.322	0.445	0.08
	Thalamus	0.060 (0.050, 0.070)	**< 0.0001**	**< 0.0001**	**3.38**	0.016 (0.007, 0.025)	**< 0.0001**	**0.0001**	**1.07**	−0.044 (−0.053, −0.034)	**< 0.0001**	**< 0.0001**	**−2.36**
ODI	Amygdala	−0.000 (−0.021, 0.021)	0.999	> 0.999	0.51	0.005 (−0.014, 0.024)	0.812	> 0.999	0.15	0.005 (−0.016, 0.026)	0.825	> 0.999	0.09
	Caudate	0.010 (−0.009, 0.029)	0.409	0.593	0.49	−0.010 (−0.028, 0.007)	0.345	0.611	−0.45	−0.021 (−0.040, −0.002)	**0.030**	**0.044**	**−0.77**
	Hippocampus	0.012 (−0.007, 0.031)	0.287	0.392	0.66	0.001 (−0.017, 0.018)	0.997	> 0.999	0.03	−0.011 (−0.031, 0.008)	0.326	0.433	−0.43
	Pallidum	0.004 (−0.022, 0.031)	0.917	> 0.999	0.15	0.008 (−0.017, 0.033)	0.746	> 0.999	0.14	0.003 (−0.024, 0.030)	0.955	> 0.999	0.02
	Putamen	0.001 (−0.015, 0.016)	0.996	> 0.999	0.30	−0.015 (−0.029, −0.000)	0.**043**	0.084	−0.51	−0.015 (−0.031, 0.000)	0.051	0.093	−0.68
	Thalamus	0.012 (0.004, 0.021)	**0.002**	**0.002**	**1.07**	−0.006 (−0.014, 0.002)	0.193	0.233	−0.42	−0.018 (−0.026, −0.010)	**< 0.0001**	**< 0.0001**	**−1.39**
MK	Amygdala	0.014 (−0.012, 0.041)	0.400	0.578	0.45	−0.008 (−0.033, 0.017)	0.703	> 0.999	−0.30	−0.023 (−0.050, 0.004)	0.108	0.187	−0.71
	Caudate	−0.008 (−0.061 0.046)	0.938	> 0.999	−0.05	0.005 (−0.045, 0.055)	0.971	> 0.999	0.09	0.012 (−0.041, 0.066)	0.845	> 0.999	0.13
	Hippocampus	0.065 (0.044, 0.086)	**< 0.0001**	**< 0.0001**	**1.87**	0.013 (−0.006, 0.033)	0.244	0.310	0.22	−0.051 (−0.072, −0.030)	**< 0.0001**	**< 0.0001**	**−1.37**
	Pallidum	0.136 (0.075, 0.198)	**< 0.0001**	**< 0.0001**	**1.07**	0.041 (−0.017, 0.100)	0.209	0.316	0.05	−0.095 (−0.157, −0.033)	**0.0001**	**0.0009**	**−0.84**
	Putamen	0.055 (0.007, 0.102)	**0.020**	**0.022**	**0.57**	−0.003 (−0.047 0.041)	0.971	> 0.999	−0.21	−0.058 (−0.105, −0.010)	**0.013**	**0.014**	**−0.80**
	Thalamus	0.075 (0.051, 0.098)	**< 0.0001**	**< 0.0001**	**1.86**	0.026 (0.004, 0.047)	**0.016**	**0.017**	**0.53**	−0.049 (−0.072, −0.025)	**< 0.0001**	**< 0.0001**	**−1.39**

Abbreviations: CI = Tukey–Kramer corrected and least squares means adjusted confidence intervals; *d* = Cohen's d effect size; L = left; MK = mean kurtosis; NDI = neurite density index; ODI = orientation dispersion index; *p* = Tukey–Kramer corrected *p*‐value from pairwise comparison between two groups; *p* (corr) = Bonferroni corrected *p*‐value; R = right; ROI = region‐of‐interest; SE = standard error.

ODI demonstrated the least sensitivity to changes of the three measures investigated, and the results did not show a clear pattern across the groups. We found evidence of decreased ODI in NF1 compared with NS in the caudate (estimate 0.021 [95% CI: 0.002, 0.040], *p* = 0.044, *d =* 0.77) and thalamus (estimate 0.018 [95% CI: 0.010, 0.026] *p* < 0.0001, *d* = 1.39).

The pattern of MK results was like NDI where NF1 < NS < TD. Lower MK was found in NF1 compared with TD in the hippocampus, pallidum, thalamus (all *p* < 0.0001), and putamen (*p* = 0.22). When comparing TD and NS, lower MK was found in NS compared with TD in the thalamus only (*p* = 0.017). MK was lower in NF1 compared with NS in the hippocampus (*p* < 0.0001), pallidum (*p* = 0.0009), putamen (*p* = 0.014), and thalamus (*p* < 0.0001).

### 3.3 Tract‐Based Analysis of NDI, ODI, and MK

**FIGURE 3:**
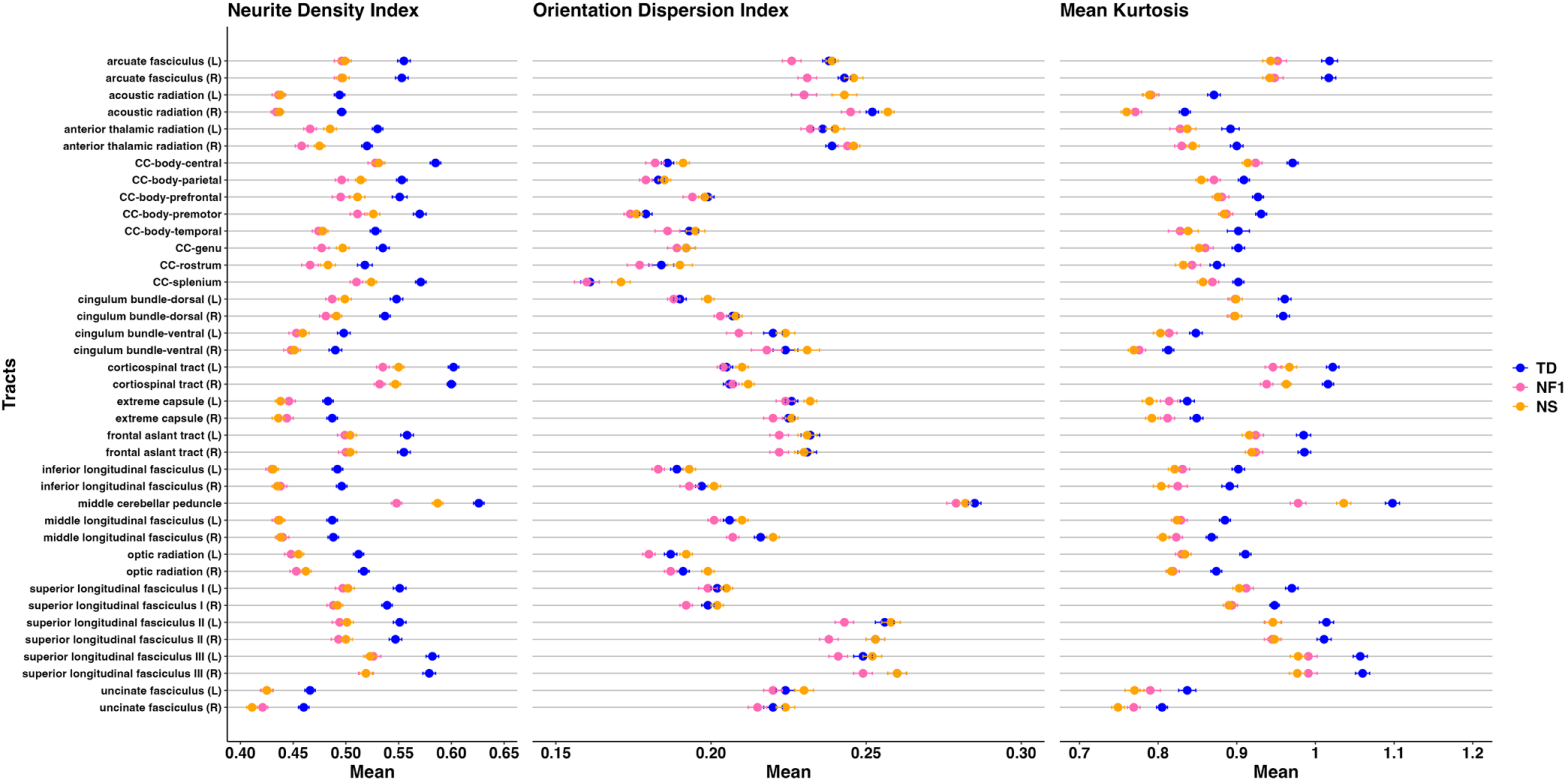
Average values and standard errors in each tract for neurite density index (NDI), orientation dispersion index (ODI), and mean kurtosis (MK). Data points represent least square means and standard errors. CC = corpus callosum; CI = confidence interval; L = left; NF1 = neurofibromatosis type 1; NS = Noonan syndrome; R = right; TD = typical developing.

**FIGURE 4:**
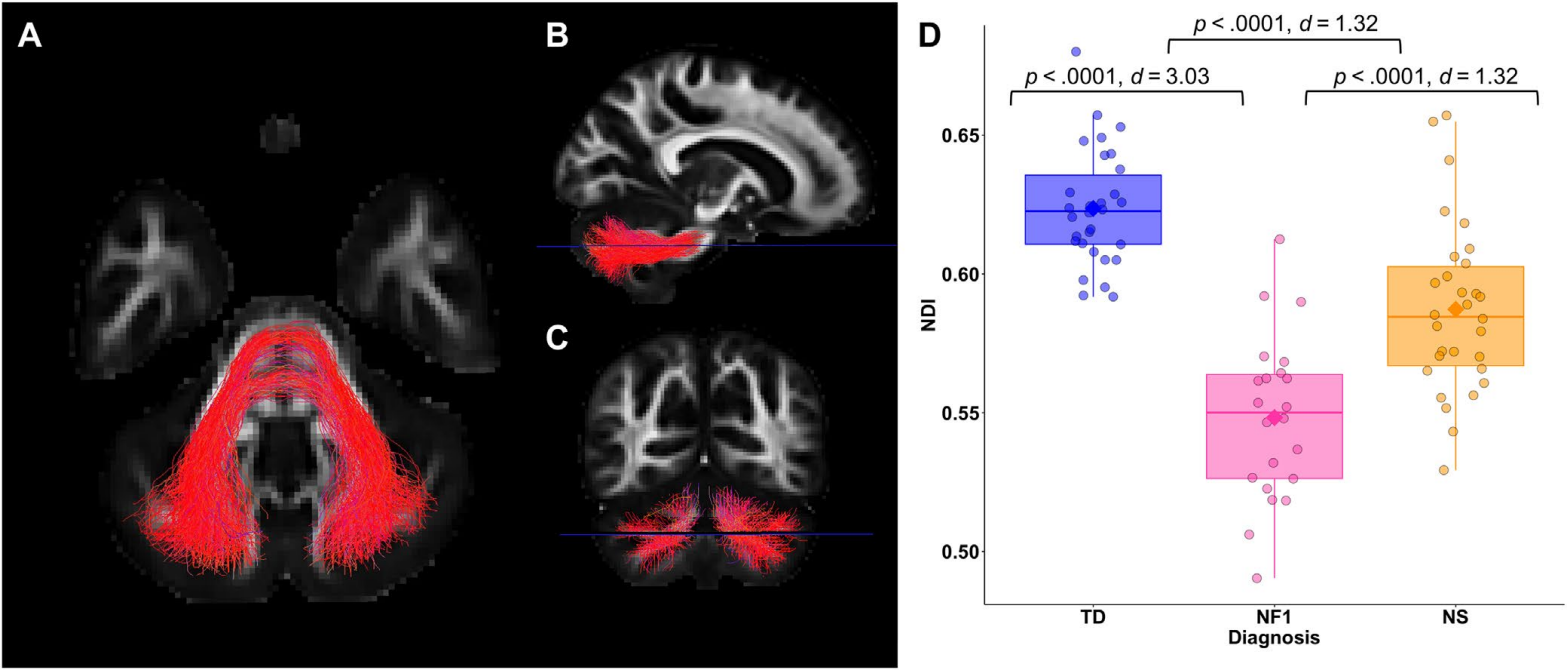
The neurite density index (NDI) in the middle cerebellar peduncle is significantly different between all three groups (NF1, NS, TD): (A) axial, (B) sagittal, and (C) coronal views of the middle cerebellar peduncle; and (D) boxplots of the NDI values in the middle cerebellar peduncle in each group. Blue line (in B and C) indicates location of slice pictured in (A). NF1 = neurofibromatosis type 1; NS = Noonan syndrome; TD = typical developing.

The number of participants included in analysis for each tract is shown in Table S3. In each of the 39 white matter tracts, we found lower NDI values in NF1 and NS compared to TD (all *p* < 0.0001) (Figures 3 and S4). The means and confidence intervals for each tract are shown in Table S4. The largest effects were found between TD and NF1 in the middle cerebellar peduncle (an estimated difference of 0.078 [95% CI: 0.060, 0.095], *p* < 0.0001, *d* = 3.03, Figure 4), left optic radiation (estimate 0.064 [95% CI: 0.046, 0.082], *p* < 0.0001, *d* = 2.05), left corticospinal tract (estimate 0.067 [95% CI: 0.050, 0.085], *p* < 0.0001, *d* = 2.08), and right corticospinal tract (estimate 0.068 [95% CI: 0.051, 0.084], *p* < 0.0001, *d* = 2.20). Only one tract showed evidence of differences between the clinical groups. Lower NDI was found in NF1 compared with NS in the middle cerebellar peduncle (estimate 0.038 [95% CI: 0.021, 0.056], *p* < 0.0001, *d* = 1.32, Figure 4).

Lower ODI was found in NF1 compared to TD in nine of the tracts; in TD compared with NS in three tracts; and in NF1 compared with NS in 18 tracts. The largest effects (Cohen's *d* > 1) were found between TD and NF1 in two tracts, and between NF1 and NS in six tracts. The right arcuate fasciculus (estimate 0.012 [95% CI: 0.003, 0.021], *p* = 0.0056, *d* = 1.00) and right superior longitudinal fasciculus II (estimate 0.014 [95% CI: 0.005, 0.024], *p* = 0.0021, *d* = 1.14) were lower in ODI in NF1 relative to TD. Comparisons between NF1 and NS showed reduced ODI in NF1 in the right arcuate fasciculus (estimate 0.015 [95% CI: 0.006, 0.024], *p* = 0.0008, *d* = 1.06), right middle longitudinal fasciculus (estimate 0.013 [95% CI: 0.006, 0.020], *p* = 0.0001, *d* = 1.26), left cingulum bundle‐dorsal (estimate 0.011 [95% CI: 0.004, 0.019], *p* = 0.0020, *d* = 1.01), left superior longitudinal fasciculus II (estimate 0.015 [95% CI: 0.005, 0.025], *p* = 0.0011, *d* = 1.06), left optic radiation (estimate 0.013 [95% CI: 0.006, 0.020], *p* = 0.0002, *d* = 1.16), and right optic radiation (estimate 0.012 [95% CI: 0.005, 0.020], *p* = 0.0006, *d* = 1.07).

Like NDI, MK was lower in NS compared to TD in all 39 tracts (*p* < 0.01). MK was also lower in NF1 compared with TD in all tracts except the rostrum of the corpus callosum and the left extreme capsule. Comparisons between NF1 and TD showed the greatest effects were in the middle cerebellar peduncle (estimate 0.119 [95% CI: 0.088, 0.151], *p* < 0.0001, *d* = 2.34), left optic radiation (estimate 0.081 [95% CI: 0.054, 0.108], *p* < 0.0001, *d* = 1.87), and right corticospinal tract (estimate 0.077 [95% CI: 0.052, 0.103], *p* < 0.0001, *d* = 1.73). The only difference between the clinical groups was found also in the middle cerebellar peduncle where MK was lower in NF1 compared to NS (estimate 0.057 [95% CI: 0.026, 0.089], *p* < 0.0001, *d* = 1.15).

Given the similar findings in NDI and MK, we tested the associations between these parameters using Pearson correlations. The results suggest the parameters are highly positively correlated, particularly in the white matter tracts. For example, MK and NDI were positively correlated in the middle cerebellar peduncle (*R* = 0.81, *p* < 0.001) (Figure S5).

### 3.4 Multivariate Analysis

**FIGURE 5:**
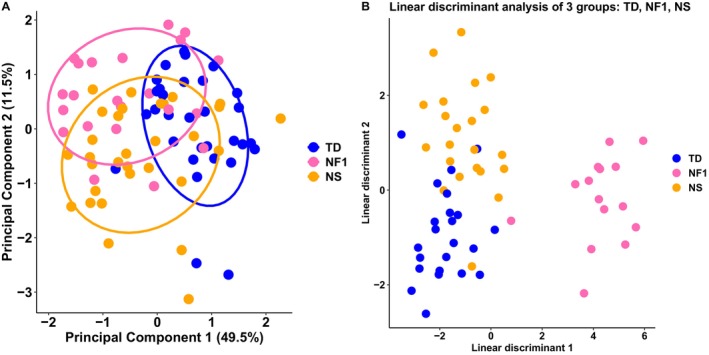
Multivariate analysis. (A) Biplot of first and second principal components generated from principal components analysis of tract data. The values of the datapoints represent loadings on each principal component while colors indicate group membership (blue for TD, pink for NF1, and orange for NS). (B) Scatterplot showing separation between groups TD (blue), NF1 (pink), and NS (orange) following linear discriminant analysis of subcortical ROI data. NDI = neurite density index; NF1 = neurofibromatosis type 1; NS = Noonan Syndrome; ROI = region‐of‐interest; TD = typical developing.

To complement the univariate results, values for each white matter tract were entered into a principal components analysis. The first component alone represented 49.0% of the variance with similar contributions from each tract (Figures 5A and S6). The second component represented 11.6% of the variance (cumulative variance of 60.6%) again with similar contributions from each tract. The eigenvalues for the first 10 principal components are detailed in Table S5. A biplot representation of the loadings in PC1 and PC2 suggest some discrimination between TD and the clinical groups (NF1, NS). Subjects in the clinical groups generally have lower values on PC1 compared to subjects in the TD group. Given that NDI and MK values contribute the most to PC1, we can infer that lower values of NDI and MK are generally found in clinical groups compared to TD. Upon visual inspection of the biplot with respect to PC2, subjects in the clinical and TD groups appear to have similar loadings. ODI values contributed the most to PC2, suggesting the groups may have similar ODI values. These inferences consolidate our pattern of univariate results.

As the univariate results showed several differences between clinical groups in the subcortical regions, we tested the ability of the subcortical diffusion values to classify the subjects into groups using linear discriminant analysis. The discriminant functions classified participants with NF1, TD, and NS with 98%, 85%, and 84% accuracy respectively. Two linear discriminant functions were generated, LD1 and LD2 (Table S6). For LD1, NDI in the thalamus contributed the most with a weighting of −1.92. Following the leave‐one‐out cross‐validation, the accuracy of the model in classifying NF1 remained high at 97.2% though there were drops in accuracy for TD and NS classifications to 71% and 70% respectively. Precision, sensitivity, specificity, and accuracy percentages in training and following cross‐validation are shown in Table S7. The highest rates were found for the NF1 group. Following cross‐validation, the precision, sensitivity, and specificity of NF1 classification were 96%, 96%, and 98.4%, respectively. Precision, sensitivity, specificity, and accuracy of TD and NS classification were similar, with values between 61% and 79%. Figures 5B and S6 show that the NF1 observations can be separated from TD and NS observations, however TD and NS observations show overlap.

## 4 Discussion

Development of effective treatments for NDDs is hindered by the heterogeneity of the disorders and limited understanding of the underlying neurobiology. This study utilized a genetics‐first approach to investigate the potential of advanced DWI techniques to elucidate the microstructural abnormalities existent in the brains of children with NF1 and NS. Analysis of white matter tracts found widespread differences between TD and the clinical groups. The middle cerebellar peduncle showed differences between NF1 and NS in both NDI and MK (*p* < 0.001). Principal components analysis confirmed that the clinical groups differ most from TD in white matter tract‐based NDI and MK, whereas ODI values appear similar across the groups. The subcortical regions showed several differences between NF1 and NS, to the extent that a linear discriminant analysis could classify participants with NF1 an accuracy rate of 97%. These results may have important implications for future neuroimaging research in clinical populations with NDDs. NODDI and DKI demonstrated the ability to image microstructure unique to NF1 and NS, suggesting the specificity of these tools may be valuable for improving understanding of brain microstructure particularly through the translation of pre‐clinical cellular findings to human clinical populations.

Prior diffusion tensor imaging studies on children with NF1 and NS found widespread reduced fractional anisotropy and increased mean diffusivity in both conditions (Siqueiros‐Sanchez et al. 2024; Fattah et al. 2021; Tam et al. 2021). Fractional anisotropy and mean diffusivity are sensitive to disruptions in microstructural integrity; however, they are also nonspecific measures and difficult to interpret in voxels with complex microstructural properties (e.g., crossing fibers) (Wheeler‐Kingshott and Cercignani 2009). Preclinical studies suggest microstructural differences between NF1 and NS (Gauthier et al. 2007; Lee et al. 2010), however, previous neuroimaging studies in the clinical populations yielded largely identical results in both conditions (Siqueiros‐Sanchez et al. 2024; Fattah et al. 2021; Tam et al. 2021). Our results, particularly in the subcortical regions and the middle cerebellar peduncle, suggest microstructural abnormalities that are unique to each condition. Use of diffusion models with greater specificity, NODDI and DKI, enabled improved elucidation of the brain structure in clinical populations with NF1 and NS.

Our observations in NF1 and NS are in alignment with pre‐clinical evidence. In NF1, proliferation of astrocytes may disturb the spacing between axons and drive a decrease in observed NDI and MK (Gutmann et al. 1999). In NS, observed reductions in NDI and MK are likely due to defects in the oligodendrocyte lineage and the consequent reduction in myelinated axons (Ehrman et al. 2014). Diffusion parameters in the subcortical regions seemed to distinguish NF1 and NS more so than in the white matter tracts, suggesting the grey matter may be the major source of differences between the two conditions. At a cellular level, it is not yet clear why grey matter may be responsible for between‐group differences in NF1 and NS. A previous pre‐clinical study of NF1 found astrocyte proliferation restricted to the grey matter (Zhu et al. 2001), though more recent work indicates astrocyte proliferation in both white and grey matter (Zhu et al. 2005).

The thalamus, a primarily grey matter region, showed lower values of NDI, MK, and ODI in NF1 compared to NS. NDI and MK in the thalamus also contributed the strongest weighting to the linear discriminant function. The thalamus has previously been highlighted in NF1 due to the presence of T2‐hyperintensities (Calvez et al. 2020). However, our sensitivity analysis showed that even when all participants with T2‐hyperintensities were excluded, the significant results in the thalamus remain. Other studies have also demonstrated that alterations to DWI parameters persist regardless of the presence of T2‐hyperintensities (Baudou et al. 2020). The presence of T2‐hyperintensities therefore is not the sole influence on differences between NF1 and NS in the thalamus. Further research is needed to understand the effects of NF1 and NS on grey matter. Future studies may consider use of diffusion models such as Soma and Neurite Density Imaging (SANDI), which might provide deeper insight into grey matter microstructure (Palombo et al. 2020).

Of the white matter tracts, the middle cerebellar peduncle emerged as the only tract where NDI and MK were both lower in NF1 compared with NS. The middle cerebellar peduncle is the major afferent pathway to the cerebellum, a region often implicated in NF1 due to the presence of T2‐hyperintensities (Billiet et al. 2014). This tract is vulnerable to abnormalities, for example myelin abnormalities and intra‐myelinic edema—both of which have been implicated in NF1 (Baudou et al. 2020; Morales and Tomsick 2015). Future research may further examine the middle cerebellar peduncle to understand the importance of its role in NDDs.

The strengths of this study lie in the genetics‐first approach and application of advanced DWI techniques to NDDs. By focusing on NDDs with specific genetic mutations, we reduced heterogeneity that typically hinders NDD studies. NF1 and NS have similar behavioral profiles and previous DWI of these clinical populations suggested comparable microstructural abnormalities (Siqueiros‐Sanchez et al. 2024). However, the specificity conferred by NODDI and DKI models enabled elucidation of microstructural differences between the conditions. The findings in this work suggest NODDI and DKI show potential for identification of treatment targets in the brain in NS and NF1. Furthermore, these diffusion models may be valuable for monitoring the effects of treatment on brain microstructure. To our knowledge, this is the first study to apply NODDI and DKI across the whole brain to clinical populations with NF1 and NS. However, this work is not without limitations. The sample sizes are small, particularly in the NF1 group (*n* = 25). Furthermore, several of the differences between TD and NS, particularly in the subcortical regions, were small. This may limit generalizability of the findings. Application of these diffusion MRI models to larger and more diverse samples would be valuable as validation of the findings in the current work. Additionally, given that DWI indirectly assesses alterations to brain microstructure, we are not able to derive with certainty the primary drivers of the differences in NDI and MK between the clinical groups. Further research on preclinical models coupled with neuroimaging is needed to assess the major influences on NDI and MK in these populations. Future studies may also explore whether the identified differences in diffusion parameters are associated with unique patterns of behavior in each condition to better understand the brain‐behavior relationships in NS and NF1.

This study provides evidence of the ability of NODDI and DKI to enhance understanding of NDDs in clinical populations particularly when used in conjunction with a genetics‐first methodology. Future research should consider the use of advanced diffusion MRI techniques to elucidate the microstructure underlying NDDs in clinical populations. Use of a genetics‐first approach may assist in identifying the distinct neurobiology in NDDs with a genetic basis, such as in autism spectrum disorder and ADHD. These approaches enable greater insight into the underlying neurobiology in NDDs and ultimately may assist in the development of more effective treatments for NDDs.

## Ethics Statement

The Stanford University School of Medicine Institutional Review Board approved all procedures in this work involving human subjects.

## Consent

Legal guardians provided written informed consent and participants aged over 7 years provided complementary written assent. All procedures comply with the ethical standards of the national and institutional committees on human experimentation and with the Helsinki Declaration of 1975, as revised in 2008.

## Conflicts of Interest

The authors declare no conflicts of interest.

## Supporting information


Data S1.

## Data Availability

The data that support the findings of this study are available from the corresponding author upon reasonable request.
