# Supplementary material for: Elucidating Microstructural Alterations in Neurodevelopmental Disorders: Application of Advanced Diffusion‐Weighted Imaging in Children With Rasopathies
Source: Hum Brain Mapp. 2024 Dec 12;45(17):e70087. doi: 10.1002/hbm.70087 (PMC11635693; doi:10.1002/hbm.70087)
Supplement: Supplementary file 1 — Data S1. [file HBM-45-e70087-s001.docx]

**Supplementary Material**

Methods and Materials

**Imaging Protocol**

All participants completed behavioral training in a mock MRI scanner to familiarize themselves with the environment and minimize motion-related artifacts (1). Structural data were collected using a whole-brain high-resolution T1-weighted magnetization-prepared rapid gradient-echo (MPRAGE) sequence: repetition time (TR)=7ms; echo time (TE)=2.83ms; inversion time (TI)=900ms; flip angle=8°; field of view (FOV)=240×240×180mm^3^; voxel size=1.0×1.0×1.2mm^3^; acquisition time (TA)=4 minutes 22 seconds. The standard Freesurfer 7.2.0 recon-all pipeline (<https://surfer.nmr.mgh.harvard.edu>) was used to preprocess the T1-weighted images. The Freesurfer reconstruction and segmentation were examined by independent raters (MR, CM) and re-run if necessary, until both raters agreed that the surfaces for each T1 met quality standards. Structural images (including T2-weighted images not described here) were visually inspected by a trained radiologist for the presence of T2-hyperintensities.

DWI data were collected using a multi-shell acquisition with *b*=500s/mm^2^ (6 directions), *b*=1000s/mm^2^ (15 directions), *b*=2000s/mm^2^ (15 directions) and *b*=3000s/mm^2^ (60 directions). A *b*=0s/mm^2^ scan was acquired between each 10 diffusion volumes (6 blip up) for subject bulk motion correction, and three additional *b*=0s/mm^2^ were collected with reverse phase encoding polarity (3 blip down) for distortion correction. Other parameters included: TR=8300ms; TE=70.5ms; FOV=240×240 mm^2^; voxel size=1.7×1.7×1.7mm^3^; slice number=75; multiband factor=3; TA=15 minutes 5 seconds. DWI data were preprocessed by MR using FSL 6.0.5 (FMRIB Analysis Group, Oxford, UK). The *topup* tool corrected susceptibility-induced distortions and the *eddy* tool corrected eddy currents-induced distortions (2,3). The *eddy* tool included options to replace outliers and correction for slice-to-volume motion (4,5). The corrected images were examined by independent raters prior to analysis (MR, JP).

**Image Analysis**

TRACULA combines distortion-corrected DWI data with T1-weighted images to reconstruct white matter tracts for each subject. 3D reconstructions of the tracts were visually examined (JP, MR) and any tracts that failed or only partially reconstructed were rerun using the *reinit* function. Tracts that failed to reconstruct or did so partially following *reinit* were excluded from analysis. Weighted means of NDI, ODI, and MK were extracted from each of the 42 tracts following the 3D tract reconstruction by TRACULA. Weighted means refer to the means of the diffusion scalars in each voxel weighted by the probability that the voxel is part of a specific tract.

**Statistical Analysis**

*Power analysis and sample size justification*

Few studies have been conducted previously using NODDI and DKI on NS and NF1, therefore the sample size justification is instead based on prior DTI studies. Power calculations were conducted using the *pwr* package in R. A prior study from our group analyzed DTI data in 17 subjects with NS and 17 TD individuals (6). At an alpha level of 0.05 and power of 0.8, the estimated minimum detectable effect size is *d*=0.99. For NF1, we found a DTI study of 18 children with NF1 compared to 26 TD individuals (7). At α=0.05 and 1-β=0.8, the estimated minimum detectable effect size is *d*=0.88. To meet a minimum detectable effect size of *d=*0.88 in the present study, we would need a sample size of at least 18 subjects per group (54 subjects total).

**Results**

**Participants**

23 participants were confirmed to have a mutation in the *Nf1* gene. The remaining participants in the NF1 group had a mutation in chromosome 17q11.2 (n=1) or presented clinically with NF1 but did not have the genetic *Nf1* mutation (n=1). Of the 31 participants in the NS group, the majority had a mutation in *PTPN11* (n=22) while the remaining participants had a mutation in *SOS1* (n=8) or *RAF1* (n=1).

**Power calculation**

Our final sample following exclusions (as detailed in the main text) included 25 NF1, 31 NS, and 32 TD. At an α=0.05 and 1-β=0.8, the minimum detectable effect size is therefore *d*=0.767.

**T2-hyperintensities**

T2-hyperintensities were identified in 18 participants with NF1. The results of a sensitivity analysis are shown in **Supplementary Table 2**.

**Tract-based analysis**

Four subjects were excluded from tract-based analysis as the tracts did not form correctly in TRACULA according to visual inspection. Three tracts were excluded from all tract-based analysis due to a low number of subjects with acceptable reconstructions: the anterior commissure (52/84 tracts [62%] excluded), left fornix (23/84 [27%] excluded), and right fornix (25/84 [30%] excluded). The number of participants included in analysis for each tract are shown in **Supplementary Table 3**.

**Supplementary Table 1. Statistical comparisons of demographic and behavioral outcomes.**

|  | **ANOVA/χ^2^** | | **Post-hoc *p*-values** | | |
| --- | --- | --- | --- | --- | --- |
|  | ***F/*χ^2^(df)** | ***p*** | **TD vs NF1** | **TD vs NS** | **NF1 vs NS** |
| **Age** | F(1,86)=2.94 | .090 |  |  |  |
| **FSIQ** | *F*(1,86)=21.3 | <.001 | **<.001** | **<.001** | .570 |
| **VIQ** | *F*(1,86)=13.1 | <.001 | **<.001** | **<.001** | .848 |
| **PIQ** | *F*(1,86)=6.61 | .012 | **<.001** | **.009** | .192 |
| **Sex (M/F)** | χ^2^(2)=2.71 | .259 |  |  |  |
| **Tanner 1** | χ^2^(6)=11.6 | .171 |  |  |  |
| **Tanner 2** | χ^2^(6)=11.8 | .067 |  |  |  |

*p*-values are not corrected for multiple comparisons.

Tanner 1 refers to Tanner Pubic Hair Scale. Tanner 2 refers to Female Breast Development/Male External Genitalia Scale.

*ANOVA= analysis of variance; FSIQ= Full-Scale Intelligence Quotient; NF1= neurofibromatosis type 1; NS= Noonan syndrome; PIQ= Performance Intelligence Quotient; TD= typical developing; VIQ= Verbal Intelligence Quotient.*

**Supplementary Table 2. Comparison of *t*-test *p*-values using different methods to account for T2 hyperintensities in subcortical regions.**

|  |  | **TD – NF1 *p*** | | | **TD – NS *p*** | | | **NF1 – NS *p*** | | |
| --- | --- | --- | --- | --- | --- | --- | --- | --- | --- | --- |
|  | **ROI** | **None** | **Covariate** | **Exclude** | **None** | **Covariate** | **Exclude** | **None** | **Covariate** | **Exclude** |
| **NDI** | **Amygdala** | **<.001** | **<.001** | **<.001** | .067 | .067 | .054 | **<.001** | .008 | **.009** |
|  | **Caudate** | **<.001** | .164 | .164 | .119 | .088 | .098 | .113 | .899 | .879 |
|  | **Hippocampus** | **<.001** | **<.001** | **<.001** | **.012** | **.006** | **.007** | **<.001** | **<.001** | **<.001** |
|  | **Pallidum** | **<.001** | **.030** | **.028** | **.021** | **.003** | **.002** | **<.001** | .929 | .938 |
|  | **Putamen** | .115 | .985 | .984 | **.004** | **.003** | **.003** | .505 | .057 | .056 |
|  | **Thalamus** | **<.001** | **<.001** | **<.001** | **<.001** | **<.001** | **<.001** | **<.001** | **<.001** | **<.001** |
| **ODI** | **Amygdala** | .942 | .689 | .686 | .727 | .825 | .808 | .918 | .463 | .448 |
|  | **Caudate** | .175 | .911 | .908 | .473 | .241 | .252 | **.015** | .323 | .330 |
|  | **Hippocampus** | .148 | .828 | .824 | .974 | .999 | .989 | .236 | .819 | .873 |
|  | **Pallidum** | **<.001** | .762 | .757 | .137 | .716 | .787 | **<.001** | .462 | .503 |
|  | **Putamen** | **.017** | .411 | .418 | .997 | .014 | .019 | **.018** | .826 | .862 |
|  | **Thalamus** | **<.001** | **.025** | **.024** | .010 | .276 | .249 | **<.001** | **.002** | **.001** |
| **MK** | **Amygdala** | .201 | .848 | .853 | .804 | .655 | .578 | .065 | .516 | .476 |
|  | **Caudate** | .969 | .986 | .987 | .958 | .996 | .984 | .872 | .975 | .963 |
|  | **Hippocampus** | **<.001** | **<.001** | **<.001** | .271 | .234 | .282 | **<.001** | **.014** | **.012** |
|  | **Pallidum** | **<.001** | .242 | .236 | .137 | .109 | .090 | **<.001** | .954 | .964 |
|  | **Putamen** | **.017** | **.044** | **.042** | .997 | .926 | .939 | **.018** | **.027** | **.027** |
|  | **Thalamus** | **<.001** | **<.001** | **<.001** | **.010** | **.011** | **.015** | **<.001** | **.009** | **.008** |

None= no method was used to account for T2-hyperintensities, n=88. Covariate= the presence of T2-hyperintensity (1=yes, 0=no) was included as a covariate in the model, n=88. T2-hyperintensities were entered as covariates in the final analysis presented in the manuscript. Exclude= 18 subjects with T2-hyperintensities were excluded from the analysis, n=70 (NS=30, NF1=8, TD=32).

*p*= Tukey-Kramer corrected *p*-value from pairwise comparison between two groups.

*L= left; R= right; ROI= region-of-interest; SE= standard error.*

**Supplementary Table 3. Number and percentages of participants included in analysis of each white matter tract.**

| **Tract** | **Total (n=84)** | **Total (%)** | **TD (n=31)** | **Total TD (%)** | **NF1 (n=22)** | **Total NF1 (%)** | **NS (n=31)** | **Total NS (%)** |
| --- | --- | --- | --- | --- | --- | --- | --- | --- |
| anterior commissure* | 32 | 38 | 12 | 38 | 7 | 32 | 13 | 42 |
| arcuate fasciculus (L) | 83 | 99 | 30 | 97 | 22 | 100 | 31 | 100 |
| arcuate fasciculus (R) | 83 | 99 | 31 | 100 | 22 | 100 | 30 | 97 |
| acoustic radiation (L) | 83 | 99 | 31 | 100 | 22 | 100 | 30 | 97 |
| acoustic radiation (R) | 83 | 99 | 31 | 100 | 22 | 100 | 30 | 97 |
| anterior thalamic radiation (L) | 84 | 100 | 31 | 100 | 22 | 100 | 31 | 100 |
| anterior thalamic radiation (R) | 82 | 98 | 30 | 97 | 22 | 100 | 30 | 97 |
| cingulum bundle-dorsal (L) | 84 | 100 | 31 | 100 | 22 | 100 | 31 | 100 |
| cingulum bundle-dorsal (R) | 84 | 100 | 31 | 100 | 22 | 100 | 31 | 100 |
| cingulum bundle-ventral (L) | 72 | 86 | 28 | 90 | 19 | 86 | 25 | 81 |
| cingulum bundle- ventral (R) | 77 | 92 | 29 | 94 | 20 | 91 | 28 | 90 |
| corpus callosum body-central | 81 | 96 | 31 | 100 | 20 | 91 | 30 | 97 |
| corpus callosum body-parietal | 84 | 100 | 31 | 100 | 22 | 100 | 31 | 100 |
| corpus callosum body-prefrontal | 84 | 100 | 31 | 100 | 22 | 100 | 31 | 100 |
| corpus callosum body-premotor | 84 | 100 | 31 | 100 | 22 | 100 | 31 | 100 |
| corpus callosum body-temporal | 83 | 99 | 31 | 100 | 22 | 100 | 30 | 97 |
| corpus callosum genu | 84 | 100 | 31 | 100 | 22 | 100 | 31 | 100 |
| corpus callosum rostrum | 77 | 92 | 30 | 97 | 21 | 95 | 26 | 84 |
| corpus callosum splenium | 83 | 99 | 31 | 100 | 22 | 100 | 30 | 97 |
| corticospinal tract (L) | 82 | 98 | 31 | 100 | 21 | 95 | 30 | 97 |
| corticospinal tract (R) | 81 | 96 | 30 | 97 | 21 | 95 | 30 | 97 |
| extreme capsule (L) | 82 | 98 | 30 | 97 | 22 | 100 | 30 | 97 |
| extreme capsule (R) | 77 | 92 | 28 | 90 | 22 | 100 | 27 | 87 |
| frontal aslant tract (L) | 83 | 99 | 31 | 100 | 22 | 100 | 30 | 97 |
| frontal aslant tract (R) | 83 | 99 | 31 | 100 | 22 | 100 | 30 | 97 |
| fornix (L)* | 61 | 73 | 26 | 84 | 13 | 59 | 22 | 71 |
| fornix (R)* | 59 | 70 | 24 | 77 | 13 | 59 | 22 | 71 |
| inferior longitudinal fasciculus (L) | 83 | 99 | 31 | 100 | 22 | 100 | 30 | 97 |
| inferior longitudinal fasciculus (R) | 83 | 99 | 31 | 100 | 22 | 100 | 30 | 97 |
| middle cerebellar peduncle | 83 | 99 | 30 | 97 | 22 | 100 | 31 | 100 |
| middle longitudinal fasciculus (L) | 81 | 96 | 31 | 100 | 22 | 100 | 28 | 90 |
| middle longitudinal fasciculus (R) | 80 | 95 | 31 | 100 | 21 | 95 | 28 | 90 |
| optic radiation (L) | 82 | 98 | 31 | 100 | 22 | 100 | 29 | 94 |
| optic radiation (R) | 81 | 96 | 29 | 94 | 22 | 100 | 30 | 97 |
| superior longitudinal fasciculus I (L) | 83 | 99 | 31 | 100 | 22 | 100 | 30 | 97 |
| superior longitudinal fasciculus I (R) | 83 | 99 | 31 | 100 | 22 | 100 | 30 | 97 |
| superior longitudinal fasciculus II (L) | 82 | 98 | 31 | 100 | 22 | 100 | 29 | 94 |
| superior longitudinal fasciculus II (R) | 82 | 98 | 31 | 100 | 22 | 100 | 29 | 94 |
| superior longitudinal fasciculus III (L) | 81 | 96 | 31 | 100 | 22 | 100 | 28 | 90 |
| superior longitudinal fasciculus III (R) | 83 | 99 | 31 | 100 | 22 | 100 | 30 | 97 |
| uncinate fasciculus (L) | 77 | 92 | 28 | 90 | 22 | 100 | 27 | 87 |
| uncinate fasciculus (R) | 80 | 95 | 31 | 100 | 22 | 100 | 27 | 87 |

*=tract excluded from analysis. Total number of subjects included in tract-based analysis n=84.

*L = left; NF1 = neurofibromatosis type 1; NS = Noonan syndrome; R = right; ROI = region-of-interest; TD = typical developing.*

**Supplementary Table 4. Least squares means and confidence intervals from analysis of white matter tracts.**

|  |  |  | **TD** | **NF1** | **NS** | **TD – NF1** | |  | | **TD – NS** | |  | **NF1 – NS** | |  |
| --- | --- | --- | --- | --- | --- | --- | --- | --- | --- | --- | --- | --- | --- | --- | --- |
|  | **Tract** | **n** | **Mean ± SE** | **Mean ± SE** | **Mean ± SE** | **Estimate (95% CI)** | ***p*** | ***p* (corr)** | **Estimate (95% CI)** | | ***p*** | ***p* (corr)** | **Estimate (95% CI)** | ***p*** | ***p* (corr)** |
| **NDI** | arcuate fasciculus (L) | 83 | 0.555 ± 0.006 | 0.496 ± 0.007 | 0.499 ± 0.006 | 0.059 (0.038, 0.081) | **<.0001** | **<.0001** | 0.056 (0.037, 0.076) | | **<.0001** | **<.0001** | -0.003 (-0.024, 0.019) | .9429 | >.9999 |
|  | arcuate fasciculus (R) | 83 | 0.553 ± 0.006 | 0.496 ± 0.007 | 0.497 ± 0.006 | 0.056 (0.035, 0.078) | **<.0001** | **<.0001** | 0.055 (0.035, 0.076) | | **<.0001** | **<.0001** | -0.001 (-0.023, 0.021) | .9924 | >.9999 |
|  | acoustic radiation (L) | 83 | 0.494 ± 0.005 | 0.436 ± 0.006 | 0.438 ± 0.005 | 0.058 (0.039, 0.076) | **<.0001** | **<.0001** | 0.056 (0.039, 0.073) | | **<.0001** | **<.0001** | -0.002 (-0.021, 0.017) | .9656 | >.9999 |
|  | acoustic radiation (R) | 83 | 0.496 ± 0.004 | 0.434 ± 0.005 | 0.437 ± 0.004 | 0.062 (0.046, 0.078) | **<.0001** | **<.0001** | 0.059 (0.044, 0.074) | | **<.0001** | **<.0001** | -0.003 (-0.019, 0.014) | .9096 | >.9999 |
|  | anterior thalamic radiation (L) | 84 | 0.530 ± 0.005 | 0.466 ± 0.006 | 0.485 ± 0.006 | 0.064 (0.044, 0.084) | **<.0001** | **<.0001** | 0.045 (0.026, 0.064) | | **<.0001** | **<.0001** | -0.019 (-0.039, 0.002) | .0780 | .0930 |
|  | anterior thalamic radiation (R) | 82 | 0.520 ± 0.005 | 0.458 ± 0.006 | 0.475 ± 0.005 | 0.061 (0.043, 0.079) | **<.0001** | **<.0001** | 0.045 (0.028, 0.062) | | **<.0001** | **<.0001** | -0.016 (-0.034, 0.002) | .0857 | .1029 |
|  | cingulum bundle-dorsal (L) | 84 | 0.548 ± 0.006 | 0.487 ± 0.006 | 0.499 ± 0.006 | 0.060 (0.040, 0.081) | **<.0001** | **<.0001** | 0.049 (0.030, 0.068) | | **<.0001** | **<.0001** | -0.011 (-0.032, 0.009) | .3897 | .5743 |
|  | cingulum bundle-dorsal (R) | 84 | 0.537 ± 0.005 | 0.481 ± 0.006 | 0.491 ± 0.005 | 0.056 (0.037, 0.075) | **<.0001** | **<.0001** | 0.046 (0.028, 0.064) | | **<.0001** | **<.0001** | -0.010 (-0.029, 0.009) | .4223 | .6347 |
|  | cingulum bundle-ventral (L) | 72 | 0.498 ± 0.006 | 0.453 ± 0.007 | 0.459 ± 0.006 | 0.045 (0.024, 0.065) | **<.0001** | **<.0001** | 0.039 (0.019, 0.058) | | **<.0001** | **<.0001** | -0.006 (-0.027, 0.015) | .7728 | >.9999 |
|  | cingulum bundle- ventral (R) | 77 | 0.490 ± 0.006 | 0.448 ± 0.007 | 0.451 ± 0.006 | 0.042 (0.021, 0.064) | **<.0001** | **<.0001** | 0.039 (0.018, 0.059) | | **<.0001** | **<.0001** | -0.004 (-0.026, 0.019) | .9144 | >.9999 |
|  | corpus callosum body-central | 81 | 0.585 ± 0.005 | 0.528 ± 0.007 | 0.531 ± 0.006 | 0.056 (0.036, 0.077) | **<.0001** | **<.0001** | 0.053 (0.034, 0.072) | | **<.0001** | **<.0001** | -0.003 (-0.024, 0.018) | .9329 | >.9999 |
|  | corpus callosum body-parietal | 84 | 0.553 ± 0.005 | 0.496 ± 0.006 | 0.514 ± 0.005 | 0.057 (0.040, 0.075) | **<.0001** | **<.0001** | 0.039 (0.023, 0.056) | | **<.0001** | **<.0001** | -0.018 (-0.036, -0.000) | **.0464** | .0535 |
|  | corpus callosum body-prefrontal | 84 | 0.551 ± 0.007 | 0.495 ± 0.008 | 0.511 ± 0.007 | 0.056 (0.032, 0.080) | **<.0001** | **<.0001** | 0.040 (0.018, 0.063) | | **.0001** | .**0001** | -0.016 (-0.040, 0.008) | .2672 | .3653 |
|  | corpus callosum body-premotor | 84 | 0.570 ± 0.006 | 0.511 ± 0.007 | 0.526 ± 0.006 | 0.060 (0.037, 0.082) | **<.0001** | **<.0001** | 0.045 (0.024, 0.066) | | **<.0001** | **<.0001** | -0.015 (-0.037, 0.008) | .2630 | .3586 |
|  | corpus callosum body-temporal | 83 | 0.528 ± 0.005 | 0.474 ± 0.006 | 0.478 ± 0.005 | 0.055 (0.037, 0.072) | **<.0001** | **<.0001** | 0.051 (0.034, 0.067) | | **<.0001** | **<.0001** | -0.004 (-0.022, 0.014) | .8478 | >.9999 |
|  | corpus callosum genu | 84 | 0.535 ± 0.006 | 0.477 ± 0.007 | 0.497 ± 0.006 | 0.058 (0.035, 0.081) | **<.0001** | **<.0001** | 0.039 (0.017, 0.060) | | **.0001** | .**0001** | -0.020 (-0.043, 0.004) | .1166 | .1438 |
|  | corpus callosum rostrum | 77 | 0.518 ± 0.007 | 0.466 ± 0.008 | 0.483 ± 0.007 | 0.053 (0.028, 0.077) | **<.0001** | **<.0001** | 0.035 (0.012, 0.058) | | **.0017** | .**0018** | -0.018 (-0.043, 0.007) | .2176 | .2885 |
|  | corpus callosum splenium | 83 | 0.571 ± 0.005 | 0.510 ± 0.006 | 0.524 ± 0.005 | 0.061 (0.042, 0.080) | **<.0001** | **<.0001** | 0.046 (0.029, 0.064) | | **<.0001** | **<.0001** | -0.014 (-0.033, 0.005) | .1986 | .2599 |
|  | corticospinal tract (L) | 82 | 0.602 ± 0.005 | 0.535 ± 0.006 | 0.550 ± 0.005 | 0.067 (0.050, 0.085) | **<.0001** | **<.0001** | 0.052 (0.036, 0.068) | | **<.0001** | **<.0001** | -0.015 (-0.033, 0.003) | .1130 | .1390 |
|  | corticospinal tract (R) | 81 | 0.600 ± 0.004 | 0.532 ± 0.005 | 0.547 ± 0.005 | 0.068 (0.051, 0.084) | **<.0001** | **<.0001** | 0.052 (0.037, 0.068) | | **<.0001** | **<.0001** | -0.015 (-0.032, 0.015) | .0825 | .0989 |
|  | extreme capsule (L) | 82 | 0.483 ± 0.005 | 0.446 ± 0.006 | 0.438 ± 0.005 | 0.036 (0.018, 0.055) | **<.0001** | **<.0001** | 0.044 (0.027, 0.062) | | **<.0001** | **<.0001** | 0.008 (-0.010, 0.027) | .5497 | .8950 |
|  | extreme capsule (R) | 77 | 0.487 ± 0.005 | 0.444 ± 0.006 | 0.436 ± 0.006 | 0.043 (0.023, 0.062) | **<.0001** | **<.0001** | 0.051 (0.032, 0.069) | | **<.0001** | **<.0001** | 0.008 (-0.012, 0.027) | .6151 | >.9999 |
|  | frontal aslant tract (L) | 83 | 0.558 ± 0.006 | 0.499 ± 0.007 | 0.504 ± 0.006 | 0.058 (0.035, 0.082) | **<.0001** | **<.0001** | 0.053 (0.032, 0.075) | | **<.0001** | **<.0001** | -0.005 (-0.029, 0.018) | .8472 | >.9999 |
|  | frontal aslant tract (R) | 83 | 0.555 ± 0.006 | 0.500 ± 0.007 | 0.504 ± 0.006 | 0.055 (0.033, 0.077) | **<.0001** | **<.0001** | 0.051 (0.031, 0.072) | | **<.0001** | **<.0001** | -0.004 (-0.026, 0.019) | .9218 | >.9999 |
|  | inferior longitudinal fasciculus (L) | 83 | 0.492 ± 0.005 | 0.430 ± 0.006 | 0.431 ± 0.005 | 0.062 (0.043, 0.081) | **<.0001** | **<.0001** | 0.061 (0.043, 0.079) | | **<.0001** | **<.0001** | -0.001 (-0.020, 0.019) | .9928 | >.9999 |
|  | inferior longitudinal fasciculus (R) | 83 | 0.496 ± 0.005 | 0.438 ± 0.006 | 0.435 ± 0.005 | 0.058 (0.040, 0.076) | **<.0001** | **<.0001** | 0.061 (0.044, 0.079) | | **<.0001** | **<.0001** | 0.003 (-0.015, 0.022) | .8982 | >.9999 |
|  | middle cerebellar peduncle | 83 | 0.626 ± 0.005 | 0.548 ± 0.005 | 0.587 ± 0.005 | 0.078 (0.060, 0.095) | **<.0001** | **<.0001** | 0.039 (0.023, 0.055) | | **<.0001** | **<.0001** | -0.038 (-0.056, -0.021) | **<.0001** | **<.0001** |
|  | middle longitudinal fasciculus (L) | 83 | 0.487 ± 0.005 | 0.436 ± 0.006 | 0.437 ± 0.005 | 0.052 (0.033, 0.070) | **<.0001** | **<.0001** | 0.050 (0.032, 0.067) | | **<.0001** | **<.0001** | -0.002 (-0.020, 0.017) | .9701 | >.9999 |
|  | middle longitudinal fasciculus (R) | 80 | 0.488 ± 0.005 | 0.440 ± 0.006 | 0.438 ± 0.005 | 0.047 (0.029, 0.065) | **<.0001** | **<.0001** | 0.050 (0.033, 0.067) | | **<.0001** | **<.0001** | 0.003 (-0.016, 0.021) | .9440 | >.9999 |
|  | optic radiation (L) | 82 | 0.512 ± 0.005 | 0.448 ± 0.006 | 0.455 ± 0.005 | 0.064 (0.046, 0.082) | **<.0001** | **<.0001** | 0.057 (0.040, 0.074) | | **<.0001** | **<.0001** | -0.007 (-0.026, 0.012) | .6338 | >.9999 |
|  | optic radiation (R) | 81 | 0.517 ± 0.005 | 0.453 ± 0.006 | 0.462 ± 0.005 | 0.065 (0.046, 0.083) | **<.0001** | **<.0001** | 0.055 (0.038, 0.072) | | **<.0001** | **<.0001** | -0.009 (-0.028, 0.009) | .4469 | .6821 |
|  | superior longitudinal fasciculus I (L) | 83 | 0.551 ± 0.006 | 0.497 ± 0.007 | 0.502 ± 0.006 | 0.054 (0.033, 0.075) | **<.0001** | **<.0001** | 0.049 (0.029, 0.068) | | **<.0001** | **<.0001** | -0.005 (-0.026, 0.016) | .8094 | >.9999 |
|  | superior longitudinal fasciculus I (R) | 83 | 0.539 ± 0.005 | 0.488 ± 0.006 | 0.492 ± 0.005 | 0.051 (0.033, 0.068) | **<.0001** | **<.0001** | 0.046 (0.030, 0.063) | | **<.0001** | **<.0001** | -0.004 (-0.023, 0.014) | .8308 | >.9999 |
|  | superior longitudinal fasciculus II (L) | 82 | 0.551 ± 0.006 | 0.494 ± 0.007 | 0.501 ± 0.006 | 0.058 (0.037, 0.078) | **<.0001** | **<.0001** | 0.051 (0.031, 0.070) | | **<.0001** | **<.0001** | -0.007 (-0.028, 0.014) | .7240 | >.9999 |
|  | superior longitudinal fasciculus II (R) | 82 | 0.547 ± 0.006 | 0.493 ± 0.007 | 0.500 ± 0.006 | 0.054 (0.033, 0.074) | **<.0001** | **<.0001** | 0.047 (0.028, 0.066) | | **<.0001** | **<.0001** | -0.006 (-0.027, 0.014) | .7389 | >.9999 |
|  | superior longitudinal fasciculus III (L) | 81 | 0.582 ± 0.006 | 0.526 ± 0.007 | 0.523 ± 0.006 | 0.056 (0.034, 0.078) | **<.0001** | **<.0001** | 0.059 (0.038, 0.080) | | **<.0001** | **<.0001** | 0.003 (-0.019, 0.026) | .9427 | >.9999 |
|  | superior longitudinal fasciculus III (R) | 83 | 0.579 ± 0.006 | 0.519 ± 0.007 | 0.519 ± 0.006 | 0.059 (0.037, 0.081) | **<.0001** | **<.0001** | 0.060 (0.039, 0.080) | | **<.0001** | **<.0001** | 0.001 (-0.022, 0.023) | .9982 | >.9999 |
|  | uncinate fasciculus (L) | 77 | 0.466 ± 0.005 | 0.425 ± 0.006 | 0.425 ± 0.005 | 0.041 (0.022, 0.060) | **<.0001** | **<.0001** | 0.041 (0.023, 0.060) | | **<.0001** | **<.0001** | 0.000 (-0.019, 0.020) | .9998 | >.9999 |
|  | uncinate fasciculus (R) | 80 | 0.460 ± 0.005 | 0.421 ± 0.005 | 0.411 ± 0.005 | 0.039 (0.023, 0.056) | **<.0001** | **<.0001** | 0.049 (0.033, 0.065) | | **<.0001** | **<.0001** | 0.009 (-0.008, 0.027) | .4125 | .6166 |
| **ODI** | arcuate fasciculus (L) | 83 | 0.238 ± 0.002 | 0.226 ± 0.003 | 0.239 ± 0.002 | 0.012 (0.003, 0.021) | **.0045** | **.0048** | -0.000 (-0.009, 0.008) | | .9898 | >.9999 | -0.013 (-0.022, -0.004) | **.0032** | **.0034** |
|  | arcuate fasciculus (R) | 83 | 0.243 ± 0.002 | 0.231 ± 0.003 | 0.246 ± 0.003 | 0.012 (0.003, 0.021) | **.0052** | .**0056** | -0.003 (-0.011, 0.006) | | .7588 | >.9999 | -0.015 (-0.024, -0.006) | **.0008** | **.0008** |
|  | acoustic radiation (L) | 83 | 0.230 ± 0.004 | 0.230 ± 0.004 | 0.243 ± 0.004 | 0.001 (-0.013, 0.014) | .9940 | >.9999 | -0.013 (-0.026, -0.001) | | **.0352** | **.0399** | -0.014 (-0.027, -0.000) | **.0451** | .0519 |
|  | acoustic radiation (R) | 83 | 0.252 ± 0.002 | 0.245 ± 0.003 | 0.257 ± 0.002 | 0.007 (-0.002, 0.015) | .1516 | .1920 | -0.006 (-0.014, 0.002) | | .2165 | .2866 | -0.012 (-0.021, -0.004) | **.0030** | **.0032** |
|  | anterior thalamic radiation (L) | 84 | 0.236 ± 0.003 | 0.232 ± 0.003 | 0.240 ± 0.003 | 0.003 (-0.006, 0.013) | .7099 | >.9999 | -0.005 (-0.014, 0.004) | | .4089 | .6097 | -0.008 (-0.018, 0.017) | .1286 | .1600 |
|  | anterior thalamic radiation (R) | 82 | 0.239 ± 0.002 | 0.244 ± 0.003 | 0.246 ± 0.002 | -0.004 (-0.012, 0.004) | .4726 | .7329 | -0.007 (-0.014, 0.001) | | .1095 | .1342 | -0.003 (-0.011, 0.006) | .7457 | >.9999 |
|  | cingulum bundle-dorsal (L) | 84 | 0.190 ± 0.002 | 0.188 ± 0.002 | 0.199 ± 0.002 | 0.002 (-0.005, 0.010) | .7250 | >.9999 | -0.009 (-0.016, -0.002) | | **.0090** | **.0098** | -0.011 (-0.019, -0.004) | **.0019** | **.0020** |
|  | cingulum bundle-dorsal (R) | 84 | 0.207 ± 0.002 | 0.203 ± 0.002 | 0.208 ± 0.002 | 0.004 (-0.002, 0.011) | .2814 | .3883 | -0.001 (-0.007, 0.006) | | .9462 | >.9999 | -0.005 (-0.012, 0.002) | .1740 | .2238 |
|  | cingulum bundle-ventral (L) | 72 | 0.220 ± 0.003 | 0.209 ± 0.004 | 0.224 ± 0.003 | 0.010 (-0.002, 0.022) | .1119 | .1377 | -0.005 (-0.016, 0.007) | | .6061 | >.9999 | -0.015 (-0.027, -0.002) | **.0155** | **.0170** |
|  | cingulum bundle- ventral (R) | 77 | 0.224 ± 0.004 | 0.218 ± 0.005 | 0.231 ± 0.004 | 0.006 (-0.008, 0.020) | .5749 | .9519 | -0.006 (-0.020, 0.007) | | .4897 | .7677 | -0.012 (-0.027, 0.002) | .1115 | .1370 |
|  | corpus callosum body-central | 81 | 0.186 ± 0.002 | 0.182 ± 0.003 | 0.191 ± 0.002 | 0.004 (-0.004, 0.013) | .4578 | .7035 | -0.005 (-0.013, 0.003) | | .3304 | .4699 | -0.009 (-0.018, -0.000) | **.0404** | **.0462** |
|  | corpus callosum body-parietal | 84 | 0.183 ± 0.002 | 0.179 ± 0.002 | 0.185 ± 0.002 | 0.004 (-0.003, 0.011) | .3641 | .5284 | -0.002 (-0.008, 0.004) | | .7089 | >.9999 | -0.006 (-0.013, 0.001) | .1009 | .1228 |
|  | corpus callosum body-prefrontal | 84 | 0.199 ± 0.002 | 0.194 ± 0.003 | 0.198 ± 0.002 | 0.005 (-0.003, 0.013) | .3480 | .5001 | 0.003 (-0.007, 0.008) | | .9960 | >.9999 | -0.004 (-0.012, 0.004) | .3984 | .5902 |
|  | corpus callosum body-premotor | 84 | 0.179 ± 0.002 | 0.174 ± 0.002 | 0.176 ± 0.002 | 0.005 (-0.001, 0.011) | .1252 | .1553 | 0.003 (-0.002, 0.009) | | .3482 | .5005 | -0.002 (-0.008, 0.004) | .7817 | >.9999 |
|  | corpus callosum body-temporal | 83 | 0.193 ± 0.003 | 0.186 ± 0.004 | 0.195 ± 0.003 | 0.007 (-0.005, 0.018) | .3373 | .4816 | -0.002 (-0.013, 0.008) | | .8653 | >.9999 | -0.009 (-0.021, 0.003) | .1554 | .1974 |
|  | corpus callosum genu | 84 | 0.192 ± 0.003 | 0.189 ± 0.003 | 0.192 ± 0.003 | 0.004 (-0.006, 0.013) | .6513 | >.9999 | 0.001 (-0.008, 0.009) | | .9890 | >.9999 | -0.003 (-0.013, 0.007) | .7377 | >.9999 |
|  | corpus callosum rostrum | 77 | 0.184 ± 0.004 | 0.177 ± 0.004 | 0.190 ± 0.004 | 0.007 (-0.006, 0.021) | .3959 | .5860 | -0.006 (-0.018, 0.007) | | .5540 | .9049 | -0.013 (-0.027, -0.001) | .0740 | .0880 |
|  | corpus callosum splenium | 83 | 0.161 ± 0.003 | 0.160 ± 0.004 | 0.171 ± 0.003 | 0.001 (-0.011, 0.013) | .9690 | >.9999 | -0.009 (-0.020, 0.002) | | .1291 | .1608 | -0.010 (-0.023, 0.002) | .1098 | .1346 |
|  | corticospinal tract (L) | 82 | 0.205 ± 0.002 | 0.204 ± 0.002 | 0.210 ± 0.002 | 0.000 (-0.007, 0.007) | .9970 | >.9999 | -0.005 (-0.012, 0.001) | | .1001 | .1218 | -0.006 (-0.013, 0.001) | .1267 | .1575 |
|  | corticospinal tract (R) | 81 | 0.206 ± 0.002 | 0.207 ± 0.002 | 0.212 ± 0.002 | -0.000 (-0.007, 0.007) | .9996 | >.9999 | -0.005 (-0.012, 0.001) | | .1379 | .1729 | -0.005 (-0.012, 0.002) | .1970 | .2575 |
|  | extreme capsule (L) | 82 | 0.226 ± 0.002 | 0.224 ± 0.003 | 0.232 ± 0.002 | 0.002 (-0.006, 0.011) | .8374 | >.9999 | -0.006 (-0.014, 0.002) | | .2163 | .2864 | -0.008 (-0.016, 0.001) | .0913 | .1103 |
|  | extreme capsule (R) | 77 | 0.225 ± 0.002 | 0.220 ± 0.003 | 0.226 ± 0.002 | 0.005 (-0.004, 0.013) | .3659 | .5319 | -0.002 (-0.010, 0.007) | | .8925 | >.9999 | -0.006 (-0.015, 0.002) | .1869 | .2428 |
|  | frontal aslant tract (L) | 83 | 0.232 ± 0.003 | 0.222 ± 0.003 | 0.231 ± 0.003 | 0.010 (0.000, 0.020) | **.0386** | .**0441** | 0.002 (-0.007, 0.011) | | .8936 | >.9999 | -0.008 (-0.018, 0.001) | .1108 | .1359 |
|  | frontal aslant tract (R) | 83 | 0.231 ± 0.003 | 0.222 ± 0.003 | 0.230 ± 0.003 | 0.009 (-0.001, 0.018) | .0732 | .0869 | 0.000 (-0.009, 0.009) | | .9964 | >.9999 | -0.009 (-0.018, 0.001) | .0944 | .1142 |
|  | inferior longitudinal fasciculus (L) | 83 | 0.189 ± 0.002 | 0.183 ± 0.002 | 0.193 ± 0.002 | 0.006 (-0.000, 0.012) | .0745 | .0885 | -0.004 (-0.010, 0.002) | | .3374 | .4817 | -0.009 (-0.016, -0.003) | **.0024** | **.0025** |
|  | inferior longitudinal fasciculus (R) | 83 | 0.197 ± 0.002 | 0.193 ± 0.003 | 0.201 ± 0.002 | 0.004 (-0.005, 0.011) | .5606 | .9192 | -0.005 (-0.013, 0.002) | | .2466 | .3329 | -0.008 (-0.017, -0.000) | **.0389** | **.0444** |
|  | middle cerebellar peduncle | 83 | 0.285 ± 0.002 | 0.279 ± 0.003 | 0.282 ± 0.002 | 0.006 (-0.002, 0.014) | .1849 | .2396 | 0.003 (-0.005, 0.010) | | .6320 | >.9999 | -0.003 (-0.011, 0.005) | .6299 | >.9999 |
|  | middle longitudinal fasciculus (L) | 81 | 0.206 ± 0.002 | 0.201 ± 0.002 | 0.210 ± 0.002 | 0.005 (-0.002, 0.012) | .1632 | .2085 | -0.004 (-0.010, 0.003) | | .3665 | .5327 | -0.009 (-0.016, -0.002) | **.0090** | **.0097** |
|  | middle longitudinal fasciculus (R) | 80 | 0.216 ± 0.002 | 0.207 ± 0.002 | 0.220 ± 0.002 | 0.009 (0.002, 0.016) | **.0054** | .**0058** | -0.004 (-0.010, 0.002) | | .3501 | .5039 | -0.013 (-0.020, -0.006) | **.0001** | **.0001** |
|  | optic radiation (L) | 82 | 0.187 ± 0.002 | 0.180 ± 0.002 | 0.192 ± 0.002 | 0.007 (0.000, 0.014) | **.0345** | .**0392** | -0.005 (-0.012, 0.001) | | .1310 | .1634 | -0.013 (-0.020, -0.006) | **.0002** | **.0002** |
|  | optic radiation (R) | 81 | 0.191 ± 0.002 | 0.187 ± 0.002 | 0.199 ± 0.002 | 0.004 (-0.004, 0.011) | .4350 | .6591 | -0.008 (-0.015, -0.001) | | **.0139** | .**0152** | -0.012 (-0.020, -0.005) | **.0005** | **.0006** |
|  | superior longitudinal fasciculus I (L) | 83 | 0.202 ± 0.002 | 0.199 ± 0.003 | 0.205 ± 0.002 | 0.003 (-0.005, 0.011) | .6149 | >.9999 | -0.003 (-0.010, 0.004) | | .5952 | .9987 | -0.006 (-0.014, 0.002) | .1693 | .2171 |
|  | superior longitudinal fasciculus I (R) | 83 | 0.199 ± 0.002 | 0.192 ± 0.002 | 0.202 ± 0.002 | 0.007 (0.000, 0.014) | **.0343** | .**0389** | -0.003 (-0.009, 0.004) | | .5759 | .9538 | -0.010 (-0.017, -0.003) | **.0028** | **.0030** |
|  | superior longitudinal fasciculus II (L) | 82 | 0.256 ± 0.003 | 0.243 ± 0.003 | 0.258 ± 0.003 | 0.013 (0.004, 0.023) | **.0039** | .**0042** | -0.002 (-0.011, 0.007) | | .8565 | >.9999 | -0.015 (-0.025, -0.005) | **.0011** | **.0011** |
|  | superior longitudinal fasciculus II (R) | 82 | 0.253 ± 0.003 | 0.238 ± 0.003 | 0.253 ± 0.003 | 0.014 (0.005, 0.024) | **.0020** | .**0021** | -0.001 (-0.010, 0.008) | | .9764 | >.9999 | -0.015 (-0.025, -0.005) | **.0014** | **.0015** |
|  | superior longitudinal fasciculus III (L) | 81 | 0.249 ± 0.003 | 0.241 ± 0.003 | 0.252 ± 0.003 | 0.008 (-0.001, 0.018) | .0890 | .1073 | -0.003 (-0.012, 0.006) | | .7130 | >.9999 | -0.011 (-0.021, -0.002) | **.0177** | **.0196** |
|  | superior longitudinal fasciculus III (R) | 83 | 0.260 ± 0.003 | 0.249 ± 0.003 | 0.260 ± 0.003 | 0.011 (0.001, 0.021) | **.0359** | .**0409** | -0.000 (-0.010, 0.009) | | .9994 | >.9999 | -0.011 (-0.021, -0.001) | **.0373** | **.0425** |
|  | uncinate fasciculus (L) | 77 | 0.224 ± 0.003 | 0.220 ± 0.003 | 0.230 ± 0.003 | 0.004 (-0.006, 0.014) | .6195 | >.9999 | -0.005 (-0.015, 0.004) | | .4016 | .5964 | -0.009 (-0.019, 0.001) | .0869 | .1046 |
|  | uncinate fasciculus (R) | 80 | 0.220 ± 0.003 | 0.215 ± 0.003 | 0.224 ± 0.003 | 0.005 (-0.006, 0.015) | .5253 | .8421 | -0.004 (-0.014, 0.007) | | .6677 | >.9999 | -0.009 (-0.020, 0.003) | .1640 | .2096 |
| **MK** | arcuate fasciculus (L) | 83 | 1.018 ± 0.010 | 0.952 ± 0.011 | 0.943 ± 0.010 | 0.066 (0.031, 0.101) | **<.0001** | <.**0001** | 0.075 (0.042, 0.107) | | **<.0001** | <.**0001** | 0.009 (-0.027, 0.044) | .8318 | >.9999 |
|  | arcuate fasciculus (R) | 82 | 1.017 ± 0.009 | 0.948 ± 0.011 | 0.942 ± 0.009 | 0.070 (0.037, 0.103) | **<.0001** | **<.0001** | 0.075 (0.044, 0.106) | | **<.0001** | **<.0001** | 0.006 (-0.028, 0.039) | .9186 | >.9999 |
|  | acoustic radiation (L) | 83 | 0.871 ± 0.008 | 0.791 ± 0.010 | 0.789 ± 0.009 | 0.080 (0.048, 0.111) | **<.0001** | **<.0001** | 0.082 (0.052, 0.111) | | **<.0001** | **<.0001** | 0.002 (-0.030, 0.034) | .9870 | >.9999 |
|  | acoustic radiation (R) | 83 | 0.834 ± 0.007 | 0.771 ± 0.008 | 0.760 ± 0.007 | 0.063 (0.038, 0.088) | **<.0001** | **<.0001** | 0.074 (0.050, 0.097) | | **<.0001** | **<.0001** | 0.010 (-0.015, 0.036) | .5969 | >.9999 |
|  | anterior thalamic radiation (L) | 83 | 0.892 ± 0.011 | 0.828 ± 0.013 | 0.837 ± 0.011 | 0.064 (0.024, 0.104) | **.0009** | **.0009** | 0.055 (0.017, 0.093) | | **.0024** | **.0025** | -0.009 (-0.050, 0.031) | .8505 | >.9999 |
|  | anterior thalamic radiation (R) | 82 | 0.900 ± 0.008 | 0.830 ± 0.009 | 0.844 ± 0.008 | 0.070 (0.041, 0.098) | **<.0001** | **<.0001** | 0.056 (0.029, 0.083) | | **<.0001** | **<.0001** | -0.014 (-0.043, 0.015) | .4851 | .7582 |
|  | cingulum bundle-dorsal (L) | 83 | 0.961 ± 0.008 | 0.898 ± 0.009 | 0.899 ± 0.008 | 0.063 (0.035, 0.091) | **<.0001** | **<.0001** | 0.062 (0.036, 0.088) | | **<.0001** | **<.0001** | -0.002 (-0.030, 0.026) | .9889 | >.9999 |
|  | cingulum bundle-dorsal (R) | 84 | 0.959 ± 0.008 | 0.897 ± 0.009 | 0.898 ± 0.008 | 0.062 (0.034, 0.091) | **<.0001** | **<.0001** | 0.061 (0.035, 0.087) | | **<.0001** | **<.0001** | -0.001 (-0.030, 0.028) | .9957 | >.9999 |
|  | cingulum bundle-ventral (L) | 72 | 0.848 ± 0.008 | 0.814 ± 0.010 | 0.803 ± 0.009 | 0.034 (0.004, 0.064) | **.0210** | **.0234** | 0.045 (0.017, 0.073) | | **.0008** | **.0008** | 0.011 (-0.020, 0.042) | .6628 | >.9999 |
|  | cingulum bundle- ventral (R) | 76 | 0.813 ± 0.007 | 0.776 ± 0.008 | 0.769 ± 0.007 | 0.037 (0.011, 0.063) | **.0030** | **.0032** | 0.044 (0.019, 0.068) | | **.0002** | **.0002** | 0.007 (-0.020, 0.033) | .8271 | >.9999 |
|  | corpus callosum body-central | 81 | 0.971 ± 0.007 | 0.924 ± 0.008 | 0.914 ± 0.007 | 0.047 (0.021, 0.073) | **.0001** | **.0001** | 0.058 (0.034, 0.082) | | **<.0001** | **<.0001** | 0.011 (-0.016, 0.037) | .6089 | >.9999 |
|  | corpus callosum body-parietal | 84 | 0.909 ± 0.007 | 0.871 ± 0.008 | 0.855 ± 0.007 | 0.038 (0.013, 0.063) | **.0013** | **.0013** | 0.054 (0.031, 0.077) | | **<.0001** | **<.0001** | 0.016 (-0.009, 0.041) | .2688 | .3679 |
|  | corpus callosum body-prefrontal | 84 | 0.927 ± 0.007 | 0.881 ± 0.009 | 0.876 ± 0.007 | 0.046 (0.019, 0.073) | **.0003** | **.0003** | 0.051 (0.026, 0.076) | | **<.0001** | **<.0001** | 0.006 (-0.022, 0.032) | .8949 | >.9999 |
|  | corpus callosum body-premotor | 84 | 0.931 ± 0.007 | 0.887 ± 0.008 | 0.884 ± 0.007 | 0.044 (0.020, 0.068) | **.0001** | **.0001** | 0.047 (0.025, 0.070) | | **<.0001** | **<.0001** | 0.003 (-0.021, 0.028) | .9507 | >.9999 |
|  | corpus callosum body-temporal | 80 | 0.902 ± 0.014 | 0.828 ± 0.015 | 0.838 ± 0.013 | 0.074 (0.025, 0.123) | **.0015** | **.0016** | 0.064 (0.018, 0.110) | | **.0039** | **.0041** | -0.010 (-0.059, 0.038) | .8671 | >.9999 |
|  | corpus callosum genu | 84 | 0.902 ± 0.008 | 0.860 ± 0.010 | 0.852 ± 0.009 | 0.042 (0.011, 0.073) | **.0045** | .**0048** | 0.049 (0.021, 0.078) | | **.0003** | **.0003** | 0.007 (-0.024, 0.038) | .8544 | >.9999 |
|  | corpus callosum rostrum | 77 | 0.875 ± 0.009 | 0.843 ± 0.011 | 0.832 ± 0.010 | 0.032 (-0.015, 0.065) | .0643 | .0757 | 0.042 (0.011, 0.074) | | **.0058** | **.0062** | 0.011 (-0.023, 0.045) | .7345 | >.9999 |
|  | corpus callosum splenium | 82 | 0.902 ± 0.007 | 0.869 ± 0.008 | 0.857 ± 0.007 | 0.034 (0.008, 0.060) | **.0076** | .0082 | 0.045 (0.021, 0.070) | | **<.0001** | **<.0001** | 0.012 (-0.015, 0.038) | .5493 | .8940 |
|  | corticospinal tract (L) | 82 | 1.022 ± 0.008 | 0.946 ± 0.010 | 0.967 ± 0.009 | 0.076 (0.044, 0.107) | **<.0001** | **<.0001** | 0.054 (0.025, 0.083) | | **<.0001** | **<.0001** | -0.021 (-0.053, 0.011) | .2548 | .3458 |
|  | corticospinal tract (R) | 80 | 1.016 ± 0.007 | 0.938 ± 0.008 | 0.963 ± 0.006 | 0.077 (0.052, 0.103) | **<.0001** | **<.0001** | 0.053 (0.029, 0.076) | | **<.0001** | **<.0001** | -0.025 (-0.050, 0.000) | .0535 | .0622 |
|  | extreme capsule (L) | 82 | 0.837 ± 0.009 | 0.814 ± 0.011 | 0.789 ± 0.009 | 0.022 (-0.11, 0.056) | .2468 | .3332 | 0.048 (0.017, 0.079) | | **.0012** | **.0013** | 0.026 (-0.008, 0.059) | .1697 | .2178 |
|  | extreme capsule (R) | 76 | 0.849 ± 0.008 | 0.812 ± 0.009 | 0.792 ± 0.008 | 0.036 (0.008, 0.065) | **.0084** | **.0091** | 0.056 (0.029, 0.084) | | **<.0001** | **<.0001** | 0.020 (-0.009, 0.048) | .2341 | .3137 |
|  | frontal aslant tract (L) | 83 | 0.985 ± 0.009 | 0.924 ± 0.010 | 0.916 ± 0.009 | 0.062 (0.029, 0.094) | **<.0001** | **<.0001** | 0.070 (0.039, 0.100) | | **<.0001** | **<.0001** | 0.008 (-0.025, 0.041) | .8346 | >.9999 |
|  | frontal aslant tract (R) | 83 | 0.986 ± 0.008 | 0.924 ± 0.009 | 0.919 ± 0.008 | 0.062 (0.003, 0.091) | **<.0001** | **<.0001** | 0.067 (0.040, 0.094) | | **<.0001** | **<.0001** | 0.005 (-0.024, 0.035) | .9067 | >.9999 |
|  | inferior longitudinal fasciculus (L) | 83 | 0.902 ± 0.008 | 0.831 ± 0.009 | 0.821 ± 0.008 | 0.072 (0.044, 0.100) | **<.0001** | **<.0001** | 0.082 (0.055, 0.108) | | **<.0001** | **<.0001** | 0.010 (-0.019, 0.038) | .7037 | >.9999 |
|  | inferior longitudinal fasciculus (R) | 83 | 0.891 ± 0.010 | 0.825 ± 0.012 | 0.804 ± 0.010 | 0.066 (0.029, 0.103) | **.0002** | **.0002** | 0.087 (0.053, 0.122) | | **<.0001** | **<.0001** | 0.021 (-0.017, 0.059) | .3786 | .5542 |
|  | middle cerebellar peduncle | 82 | 1.098 ± 0.009 | 0.978 ± 0.010 | 1.036 ± 0.009 | 0.119 (0.088, 0.151) | **<.0001** | **<.0001** | 0.062 (0.032, 0.092) | | **<.0001** | **<.0001** | -0.057 (-0.089, -0.026) | **.0001** | **.0001** |
|  | middle longitudinal fasciculus (L) | 81 | 0.885 ± 0.007 | 0.829 ± 0.008 | 0.824 ± 0.007 | 0.055 (0.029, 0.081) | **<.0001** | **<.0001** | 0.061 (0.036, 0.085) | | **<.0001** | **<.0001** | 0.005 (-0.021, 0.032) | .8768 | >.9999 |
|  | middle longitudinal fasciculus (R) | 80 | 0.868 ± 0.007 | 0.823 ± 0.008 | 0.806 ± 0.007 | 0.044 (0.020, 0.069) | **.0001** | **.0001** | 0.062 (0.039, 0.085) | | **<.0001** | **<.0001** | 0.017 (-0.008, 0.043) | .2390 | .3212 |
|  | optic radiation (L) | 82 | 0.911 ± 0.007 | 0.830 ± 0.008 | 0.834 ± 0.008 | 0.081 (0.054, 0.108) | **<.0001** | **<.0001** | 0.077 (0.052, 0.102) | | **<.0001** | **<.0001** | -0.004 (-0.031, 0.024) | .9373 | >.9999 |
|  | optic radiation (R) | 81 | 0.874 ± 0.007 | 0.819 ± 0.008 | 0.817 ± 0.007 | 0.056 (0.030, 0.081) | **<.0001** | **<.0001** | 0.057 (0.034, 0.081) | | **<.0001** | **<.0001** | 0.002 (-0.024, 0.027) | .9863 | >.9999 |
|  | superior longitudinal fasciculus I (L) | 83 | 0.970 ± 0.008 | 0.912 ± 0.009 | 0.903 ± 0.008 | 0.058 (0.030, 0.086) | **<.0001** | **<.0001** | 0.067 (0.041, 0.093) | | **<.0001** | **<.0001** | 0.010 (-0.020, 0.038) | .7368 | >.9999 |
|  | superior longitudinal fasciculus I (R) | 83 | 0.948 ± 0.006 | 0.894 ± 0.007 | 0.890 ± 0.007 | 0.054 (0.030, 0.077) | **<.0001** | **<.0001** | 0.057 (0.035, 0.079) | | **<.0001** | **<.0001** | 0.004 (-0.020, 0.027) | .9289 | >.9999 |
|  | superior longitudinal fasciculus II (L) | 83 | 1.014 ± 0.009 | 0.946 ± 0.011 | 0.946 ± 0.010 | 0.069 (0.034, 0.103) | **<.0001** | **<.0001** | 0.068 (0.036, 0.101) | | **<.0001** | **<.0001** | -0.000 (-0.035, 0.035) | .9997 | >.9999 |
|  | superior longitudinal fasciculus II (R) | 82 | - 1. 1 ± 0.009 | 0.945 ± 0.010 | 0.948 ± 0.009 | 0.065 (0.033, 0.098) | **<.0001** | **<.0001** | 0.062 (0.032, 0.093) | | **<.0001** | **<.0001** | -0.003 (-0.036, 0.030) | .9747 | >.9999 |
|  | superior longitudinal fasciculus III (L) | 82 | 1.057 ± 0.009 | 0.991 ± 0.011 | 0.978 ± 0.010 | 0.066 (0.031, 0.101) | **<.0001** | **<.0001** | 0.079 (0.045, 0.112) | | **<.0001** | **<.0001** | 0.013 (-0.023, 0.049) | .6717 | >.9999 |
|  | superior longitudinal fasciculus III (R) | 83 | 1.060 ± 0.009 | 0.991 ± 0.011 | 0.977 ± 0.010 | 0.070 (0.035, 0.104) | **<.0001** | **<.0001** | 0.084 (0.051, 0.116) | | **<.0001** | **<.0001** | 0.014 (-0.021, 0.049) | .6060 | >.9999 |
|  | uncinate fasciculus (L) | 76 | 0.837 ± 0.011 | 0.790 ± 0.013 | 0.770 ± 0.012 | 0.047 (0.006, 0.088) | **.0193** | **.0214** | 0.067 (0.028, 0.106) | | **.0003** | **.0003** | 0.020 (-0.021, 0.062) | .4808 | .7496 |
|  | uncinate fasciculus (R) | 80 | 0.805 ± 0.007 | 0.769 ± 0.008 | 0.749 ± 0.008 | 0.036 (0.011, 0.062) | **.0034** | **.0036** | 0.056 (0.032, 0.081) | | **<.0001** | **<.0001** | 0.020 (-0.006, 0.047) | .1692 | .2171 |

*CI = 95% confidence interval; L = left; NDI = neurite density index; NF1 = neurofibromatosis type 1; NS = Noonan syndrome; ODI = orientation dispersion index; p= Tukey-Kramer corrected p-value; p (corr) = Bonferroni corrected p-value; MK = mean kurtosis; R = right; TD = typical developing.*

**Supplementary Table 5. Top 10 components in principal components analysis of tracts.**

| **Component** | **Proportion of variance** | **Cumulative proportion** | **Standard deviation** |
| --- | --- | --- | --- |
| **PC1** | 0.495 | 0.495 | 7.61 |
| **PC2** | 0.115 | 0.611 | 3.68 |
| **PC3** | 0.047 | 0.658 | 2.35 |
| **PC4** | 0.032 | 0.690 | 1.94 |
| **PC5** | 0.027 | 0.717 | 1.77 |
| **PC6** | 0.023 | 0.740 | 1.66 |
| **PC7** | 0.018 | 0.758 | 1.45 |
| **PC8** | 0.016 | 0.775 | 1.39 |
| **PC9** | 0.016 | 0.791 | 1.38 |
| **PC10** | 0.015 | 0.806 | 1.33 |

**Supplementary Table 6. Training and cross-validation parameters for linear discriminant analysis of the subcortical regions.**

|  |  | | **TD (n=32)** | **NF1 (n=25)** | **NS (n=31)** |
| --- | --- | --- | --- | --- | --- |
| **Training** | | **Precision %** | 77.1 | 100 | 82.8 |
|  | | **Sensitivity %** | 84.4 | 96.0 | 77.4 |
|  | | **Specificity %** | 85.7 | 100 | 91.2 |
|  | | **Accuracy %** | 85.0 | 98.0 | 84.3 |
| **Cross-validation** | | **Precision %** | 62.5 | 96.0 | 61.3 |
|  | | **Sensitivity %** | 62.5 | 96.0 | 61.3 |
|  | | **Specificity %** | 78.6 | 98.4 | 79.0 |
|  | | **Accuracy %** | 70.5 | 97.2 | 70.1 |

*NF1 = neurofibromatosis type 1; NS = Noonan syndrome; TD = typical developing.*

**Supplementary Table 7.** **Coefficients of linear discriminant functions.**

|  |  | **Coefficients** | |
| --- | --- | --- | --- |
|  |  | **LD1** | **LD2** |
| **NDI** | **Pallidum** | 0.135 | 0.102 |
|  | **Caudate** | 0.008 | 0.078 |
|  | **Amygdala** | 0.559 | -0.117 |
|  | **Thalamus** | -1.921 | -0.768 |
|  | **Hippocampus** | -0.913 | 0.193 |
|  | **Putamen** | 0.609 | -0.735 |
| **ODI** | **Pallidum** | 0.280 | 0.055 |
|  | **Caudate** | 0.513 | 0.007 |
|  | **Amygdala** | 0.356 | -0.193 |
|  | **Thalamus** | -0.428 | 0.434 |
|  | **Hippocampus** | -0.414 | -0.075 |
|  | **Putamen** | -0.390 | 0.676 |
| **MK** | **Pallidum** | 0.338 | -0.331 |
|  | **Caudate** | 0.161 | 0.633 |
|  | **Amygdala** | 0.269 | -0.364 |
|  | **Thalamus** | -0.850 | 0.020 |
|  | **Hippocampus** | 0.036 | 0.563 |
|  | **Putamen** | -0.466 | 0.001 |

*MK = mean kurtosis; NDI = neurite density index; ODI = orientation dispersion index.*

**Supplementary References**

1. de Bie HMA, Boersma M, Wattjes MP, Adriaanse S, Vermeulen RJ, Oostrom KJ, et al. Preparing children with a mock scanner training protocol results in high quality structural and functional MRI scans. Eur J Pediatr. 2010 Sep 1;169(9):1079–85.

2. Andersson JLR, Skare S, Ashburner J. How to correct susceptibility distortions in spin-echo echo-planar images: application to diffusion tensor imaging. Neuroimage. 2003 Oct;20(2):870–88.

3. Andersson JLR, Sotiropoulos SN. An integrated approach to correction for off-resonance effects and subject movement in diffusion MR imaging. Neuroimage. 2016 Jan 15;125:1063–78.

4. Andersson JLR, Graham MS, Zsoldos E, Sotiropoulos SN. Incorporating outlier detection and replacement into a non-parametric framework for movement and distortion correction of diffusion MR images. Neuroimage. 2016 Nov 1;141:556–72.

5. Andersson JLR, Graham MS, Drobnjak I, Zhang H, Filippini N, Bastiani M. Towards a comprehensive framework for movement and distortion correction of diffusion MR images: Within volume movement. Neuroimage. 2017 May 15;152:450–66.

6. Fattah M, Raman MM, Reiss AL, Green T. PTPN11 Mutations in the Ras-MAPK Signaling Pathway Affect Human White Matter Microstructure. Cereb Cortex. 2021 Feb 5;31(3):1489–99.

7. Tam LT, Ng NN, McKenna ES, Bruckert L, Yeom KW, Campen CJ. Effects of Age on White Matter Microstructure in Children With Neurofibromatosis Type 1. J Child Neurol. 2021 Sep;36(10):894–900.

**
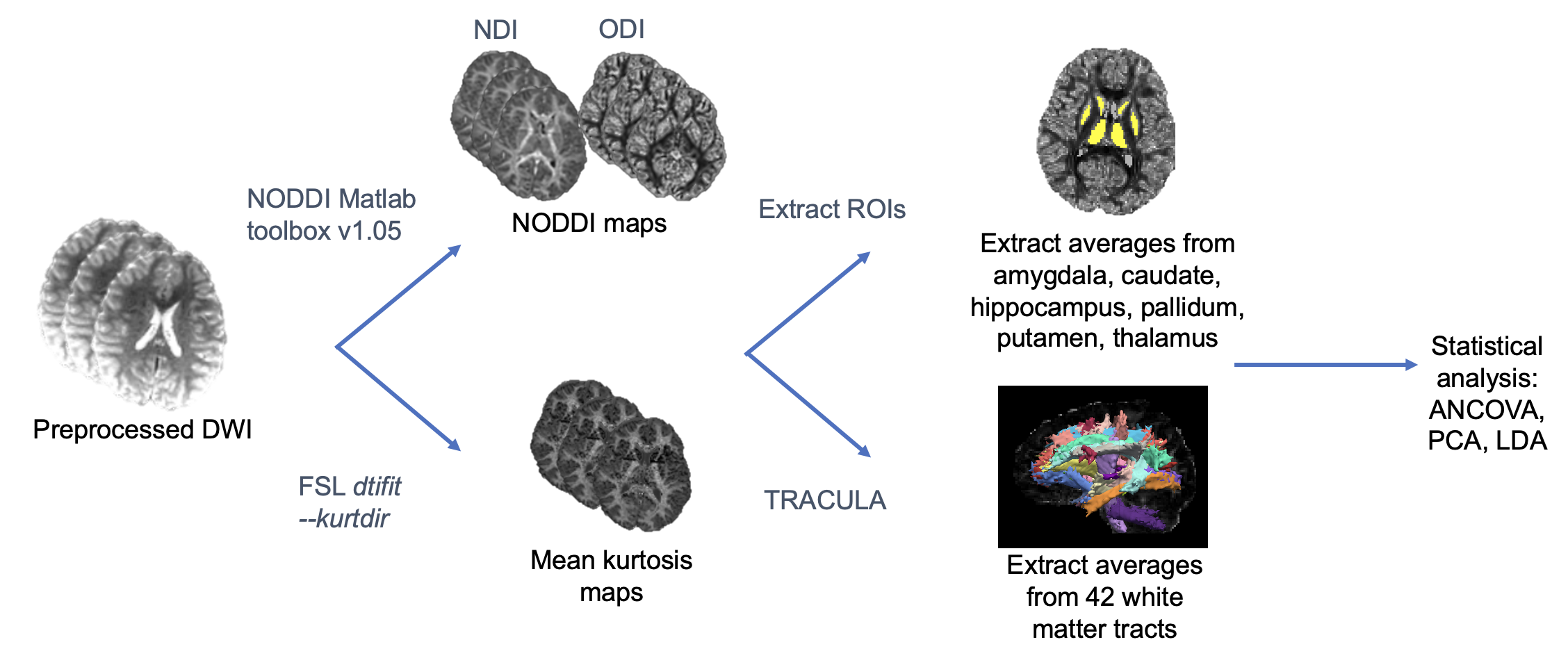
**

**Supplementary Figure 1. Flowchart of data analysis pipeline.**

*ANOVA = analysis of covariance; DWI = diffusion-weighted imaging; LDA = linear discriminant analysis; NDI = neurite density index; NODDI = neurite orientation dispersion and density imaging; ODI = orientation dispersion index; PCA = principal components analysis; ROI = region-of-interest; TRACULA = TRActs Constrained by UnderLying Anatomy.*

**
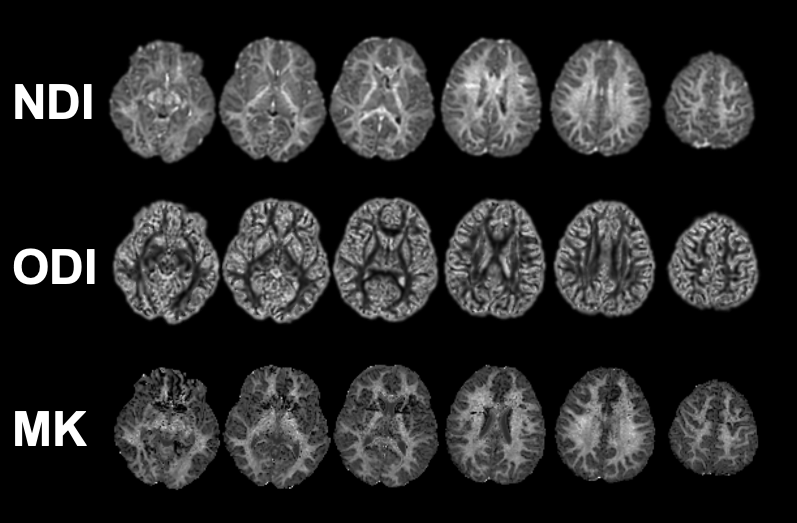
**

**Supplementary Figure 2. NDI, ODI, and MK maps selected from a single participant showing identical slices.**

*NDI = neurite density index; ODI = orientation dispersion index; MK = mean kurtosis.*

*
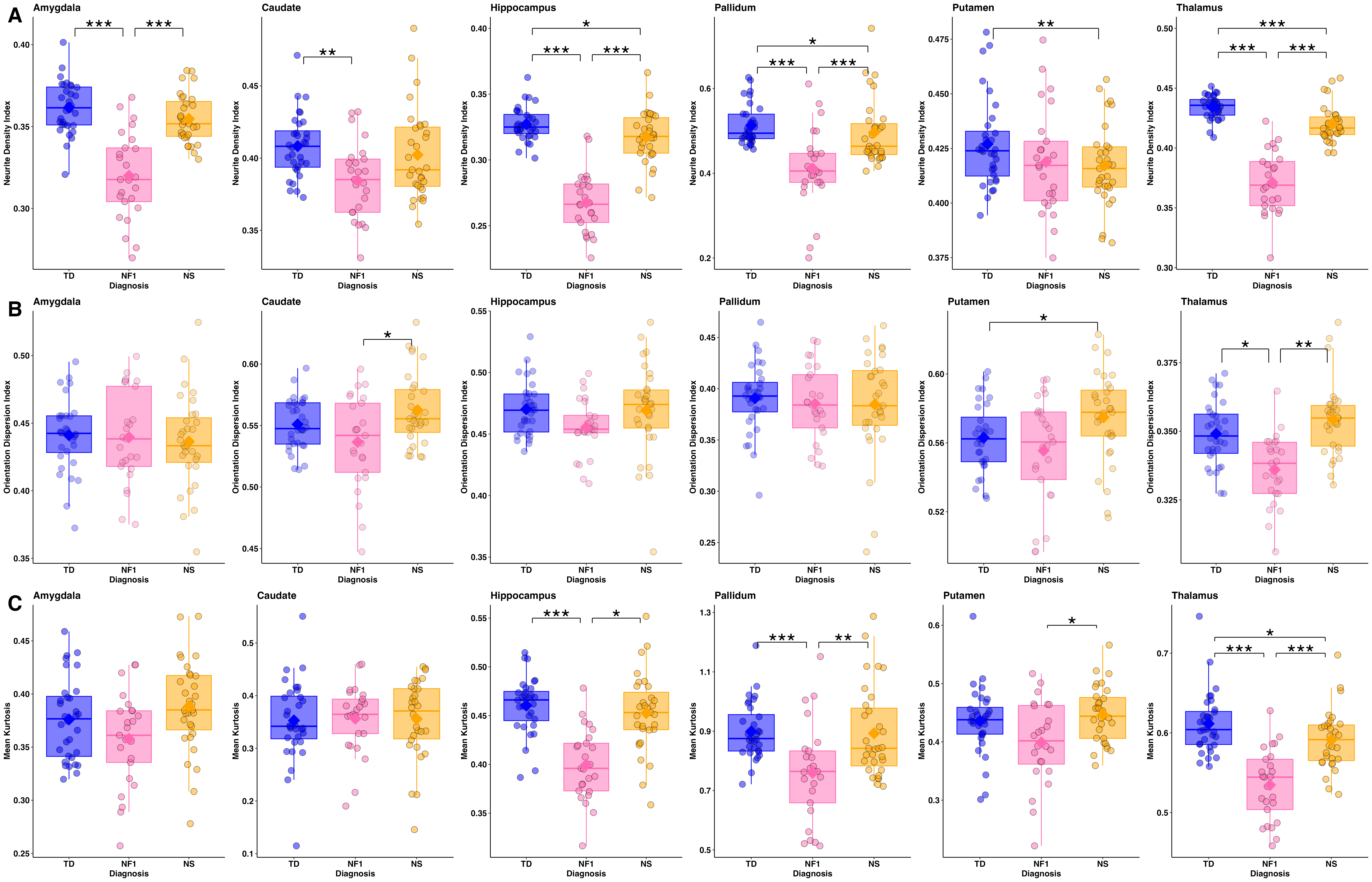
*

**Supplementary Figure 3. A) NDI, B) ODI, and C) MK in each of the subcortical regions by diagnosis TD (blue), NF1 (pink), and NS (orange).** * = *p* <.05; ** = .009 > *p* > .001; *** = *p*<.001. All *p*’s are Tukey-Kramer corrected.

*NDI = neurite density index; NF1 = neurofibromatosis type 1; NS = Noonan syndrome; ODI = orientation dispersion index; MK = mean kurtosis; TD = typical developing.*


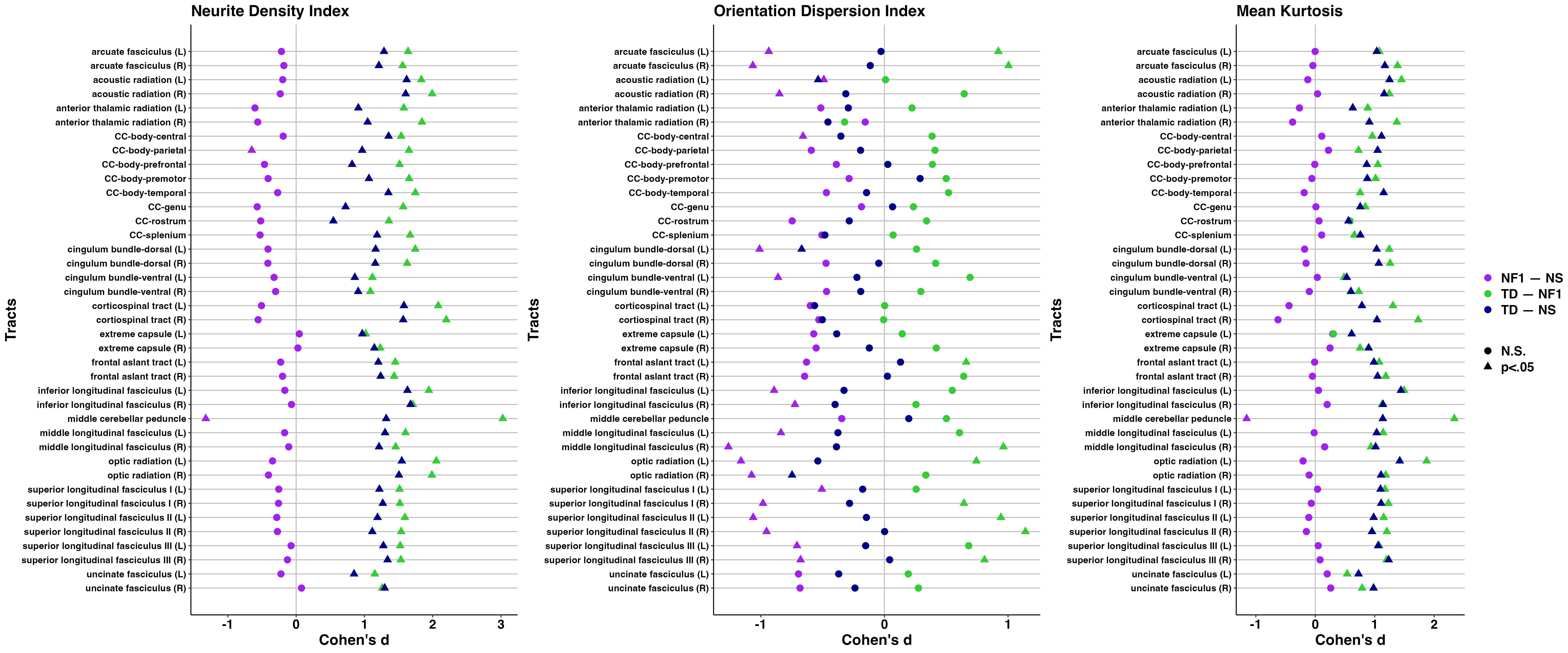


**Supplementary Figure 4. Cohen’s *d* effect sizes for the NDI, ODI, and MK comparisons in each tract.** Comparisons shown are NF1 vs NS (purple), TD vs NF1 (green), and TD vs NS (blue). Circles indicate the comparison is non-significant, whereas triangles indicate the comparison is significant at *p*<.05 Tukey-Kramer corrected.

*CC = corpus callosum; L = left; NF1 = neurofibromatosis type 1; NS = Noonan syndrome; R = right; TD = typical developing.*

**
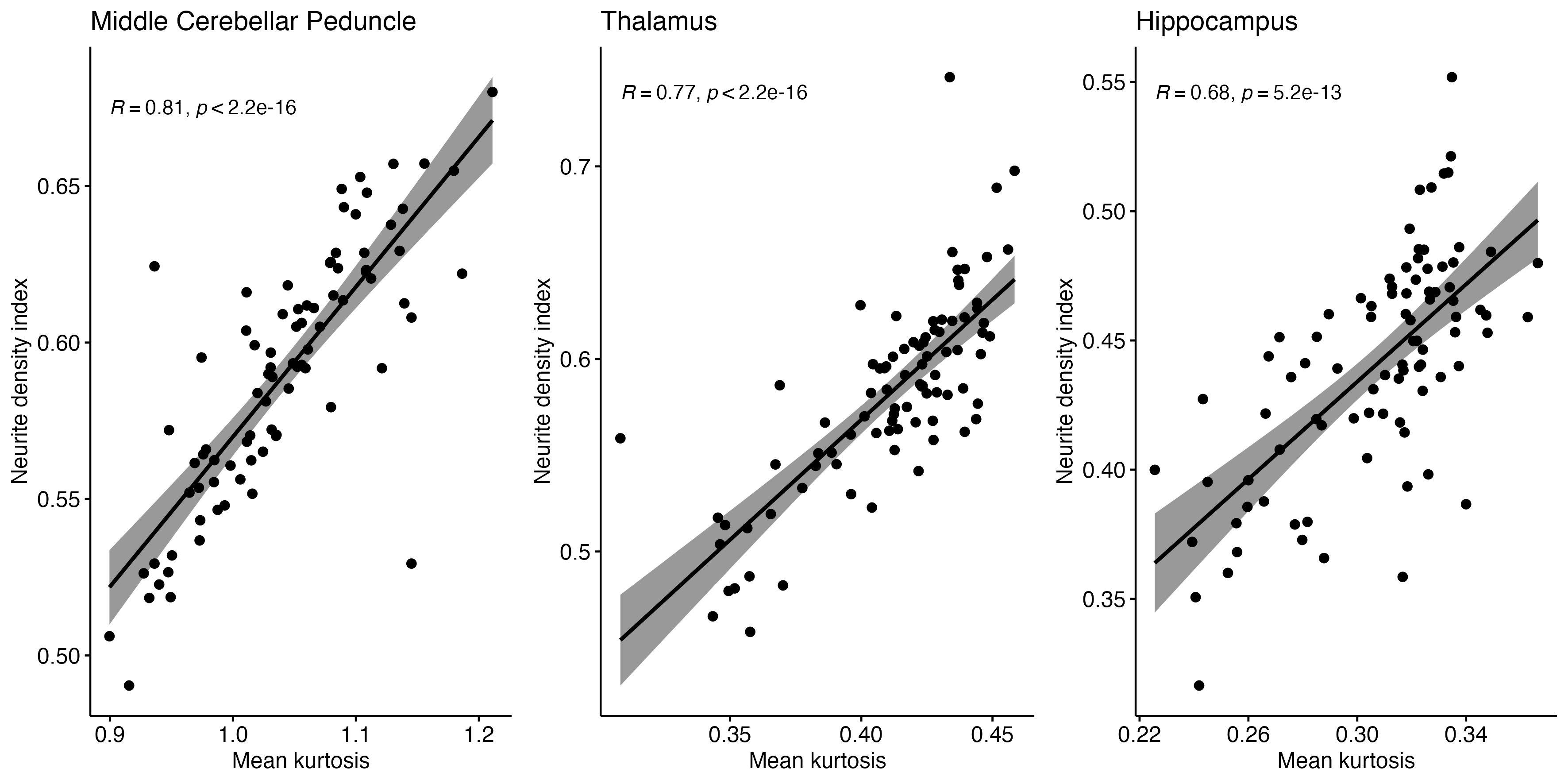
**

**Supplementary Figure 5. Neurite density index plotted against mean kurtosis values from the middle cerebellar peduncle, thalamus, and hippocampus.**

**
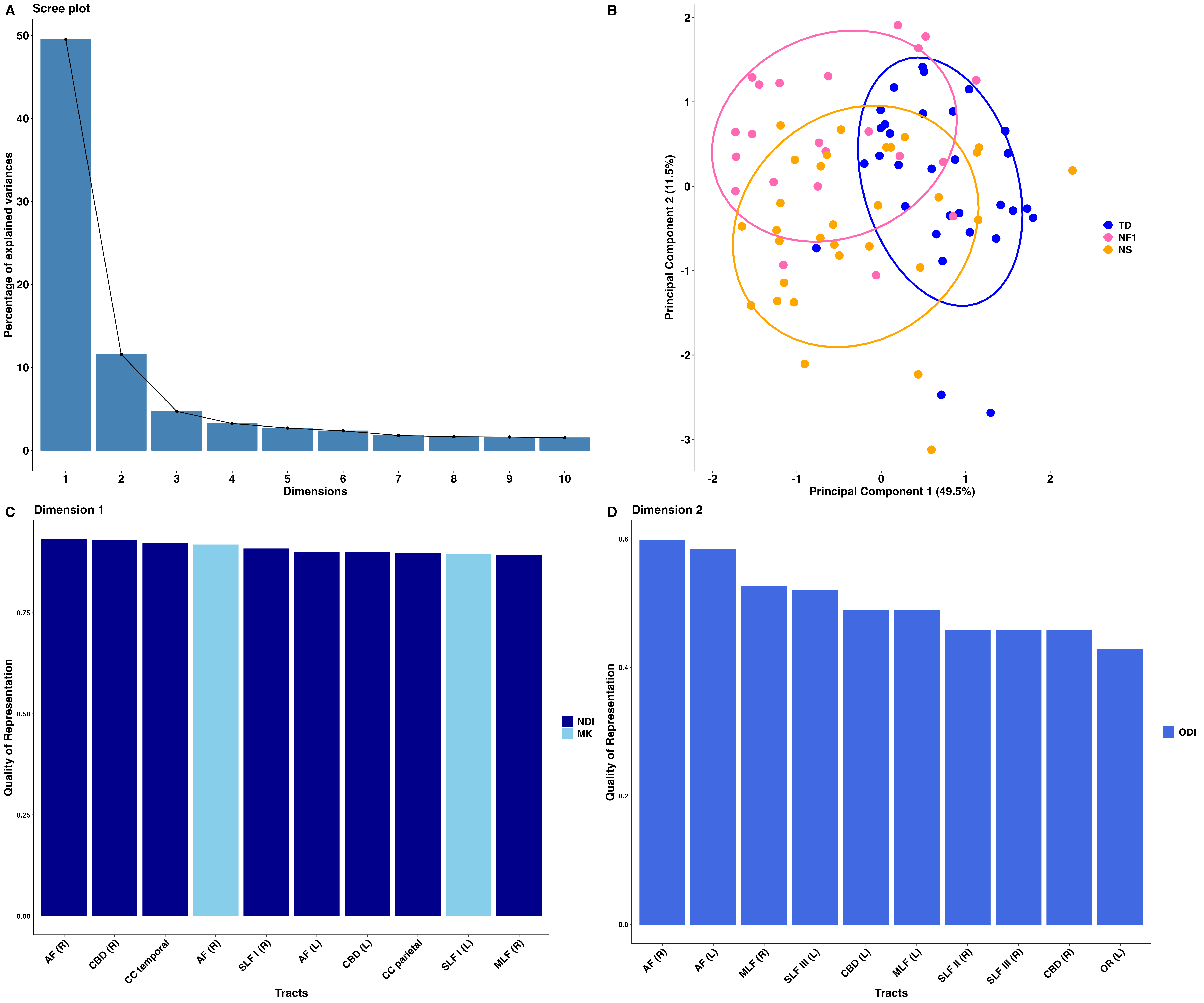
**

**Supplementary Figure 6. Principal components analysis of tract data.**

**A)** Scree plot of explained variances by each dimension. The first dimension (PC1) explains 49% of the variance in the dataset.

**B)** Biplot of first and second principal components. The values of the datapoints represent loadings on each principal component while colours indicate group membership (blue for TD, pink for NF1, and orange for NS).

**C)** Bar plot of the top 10 contributions to the first dimension (PC1). The contributions are roughly equal. Dark blue indicates NDI data and light blue indicates MK data.

**D)** Bar plot of the top 10 contributions to the second dimension (PC2). The left arcuate fasciculus contributes the most to PC2. All contributions shown are from ODI data.

*AF = arcuate fasciculus; ATR = anterior thalamic radiation; CBD = cingulum bundle-dorsal; CC = corpus callosum; CST = corticospinal tract; FAT = frontal aslant tract; ILF = inferior longitudinal fasciculus; L = left; MLF = middle longitudinal fasciculus; MK = mean kurtosis; NDI = neurite density index; ODI = orientation dispersion index; PCA = principal components analysis; R = right; SLF = superior longitudinal fasciculus; TD = typical developing*.

**
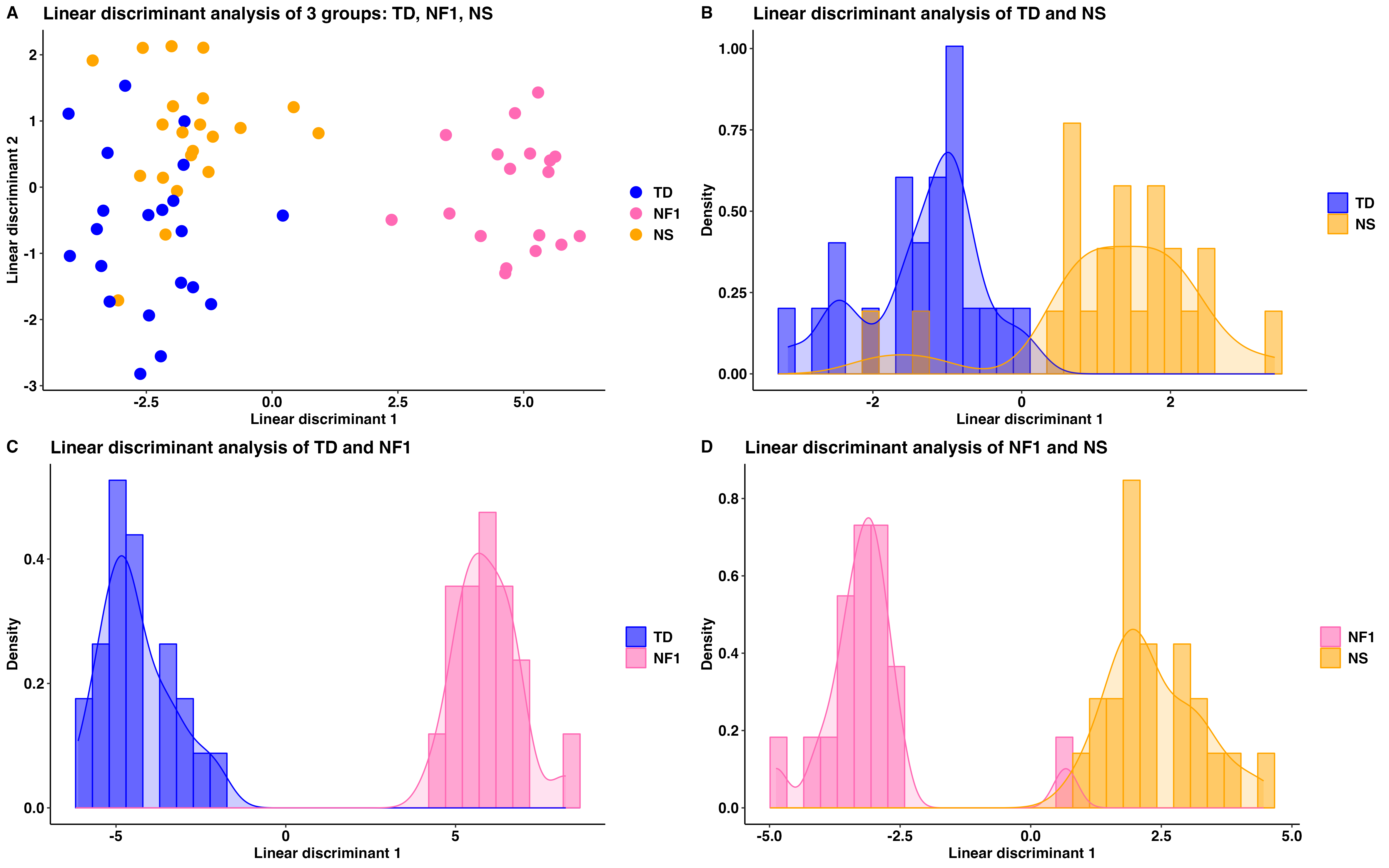
**

**Supplementary Figure 6. Linear discriminant analysis of subcortical ROI data.**

**A)** Scatterplot showing separation between groups TD (blue), NF1 (pink), and NS (orange).

**B)** Histogram showing some overlap between TD and NS distribution.

**C)** Histogram showing complete separation between TD and NF1.

**D)** Histogram showing almost complete separation between NF1 and NS.

*NDI = neurite density index; NS = Noonan Syndrome; ROI = region-of-interest; TD = typical developing.*
